# Coordinated Pre- and Postsynaptic Protein Dynamics Underlie Rapid Sema4D-Induced Inhibitory Synapse Assembly

**DOI:** 10.1523/ENEURO.0140-26.2026

**Published:** 2026-05-29

**Authors:** Zachary Pranske, Suzanne Paradis

**Affiliations:** Department of Biology, Brandeis University, Waltham, Massachusetts 02454

**Keywords:** GABAergic, inhibition, live cell imaging, Semaphorin/Plexin, synapse formation, synaptogenesis

## Abstract

Inhibitory synapse formation is poorly understood compared with excitatory synaptogenesis, in part because the molecular events underlying assembly occur asynchronously and on timescales that have been difficult to resolve. Here, we exploit the ability of Semaphorin 4D (Sema4D) to rapidly and selectively induce GABAergic synapse formation in cultured hippocampal neurons, synchronizing these events to enable direct observation of pre- and postsynaptic protein dynamics by two-channel live imaging. We find that Sema4D promotes a population-wide increase in the mobility of GAD65-containing presynaptic boutons within 20 min of treatment while postsynaptic gephyrin scaffolds are mobilized only locally in a proximity-dependent manner, consistent with a presynapse-first model of inhibitory synapse assembly. Sema4D also drives recruitment of GABA_A_Rγ2 subunits to receptor-poor postsynaptic gephyrin scaffolds within 10 min of treatment, prior to detectable changes in GAD65–gephyrin colocalization, suggesting that postsynaptic scaffolds are primed for receptor capture before alignment with a presynaptic partner. Finally, we observed new colocalization events between established gephyrin and GABA_A_R protein assemblies, suggesting that clustering of either the gephyrin scaffold or GABA_A_Rs alone is sufficient to nucleate assembly of the postsynaptic specialization. Together, these results reveal a temporally ordered, spatially constrained mechanism by which Sema4D coordinates pre- and postsynaptic protein dynamics to assemble inhibitory synapses on the timescale of minutes.

## Significance Statement

The assembly of new synaptic contacts requires precise coordination of specialized proteins in pre- and postsynaptic neurons. Inhibitory synapses, which suppress neuronal activity and are essential for circuit stability, contain distinct molecular components, yet the mechanisms governing their assembly remain poorly understood. We used Sema4D, a protein that rapidly induces inhibitory synapse formation, as a molecular tool to dissect how synaptic proteins on either side of the synaptic cleft are coordinated in space and time. Using live imaging, we show that Sema4D acts on both pre- and postsynaptic compartments to recruit synaptic proteins with spatiotemporal precision. Together, these findings define the sequence of molecular events underlying inhibitory synapse assembly and have implications for neurodevelopmental disorders in which inhibition is disrupted.

## Introduction

Synapses are the core unit of cell–cell communication in the nervous system. Early studies of excitatory, glutamatergic synapse assembly in the hippocampus revealed that synapse formation begins with the establishment of a transient contact between an axon and a dendrite (Scheiffele, 2003; Ziv and Garner, 2004). Next, mobile, preclustered protein assemblies are localized to the presynaptic compartment (Ziv and Garner, 2004; McAllister, 2007), while postsynaptic maturation is marked by accumulation of neurotransmitter receptors and scaffolding proteins (Prange and Murphy, 2001; Bresler et al., 2004). Synaptic signaling molecules such as LRRTMs, Teneurins, Neuroligin/Neurexins, and Semaphorin/Plexins are essential for both initial contact formation and subsequent recruitment of synaptic proteins (Kuzirian et al., 2013; McDermott et al., 2018; Südhof, 2018, 2021), although it remains unclear at which specific stage(s) of synapse development these molecules act.

While this sequential model of synapse assembly has been most thoroughly characterized at excitatory synapses, whether the same principles govern inhibitory synapse formation is not understood. Only a handful of studies have directly addressed the steps involved in GABAergic synapse formation using live-imaging approaches (Wierenga et al., 2008; Dobie and Craig, 2011; Kuriu et al., 2012; Villa et al., 2016; Frias et al., 2019). These studies revealed gradual protein accumulation at axon–dendrite crossings over hours but did not resolve molecular recruitment events on the order of seconds to minutes. This limitation is significant, as the earliest stages of synapse assembly are likely governed by rapid, transient molecular events, leaving a substantial gap in understanding how molecular signals acutely regulate cellular processes that transform nascent contacts into mature GABAergic synapses.

We discovered that the extracellular domain of the trans-synaptic signaling protein Semaphorin 4D (Sema4D) is sufficient to drive formation of new inhibitory synapses between hippocampal neurons within 30 min, detected by immunostaining for proteins that specifically localize to inhibitory synapses (Kuzirian et al., 2013; McDermott et al., 2018; Adel et al., 2023). These synapses become functional within 2 h as revealed by electrophysiological studies in dissociated hippocampal neurons and acute hippocampal slice preparations (Kuzirian et al., 2013; Adel et al., 2023), establishing that Sema4D-dependent effects on the organization of synaptic components are precursors to synapse formation itself.

Both loss-of-function (Paradis et al., 2007; Raissi et al., 2013) and gain-of-function studies (Kuzirian et al., 2013; McDermott et al., 2018; Adel et al., 2023) demonstrate that Sema4D specifically regulates GABAergic synapse formation without affecting glutamatergic synapses, making it a uniquely selective and temporally precise signaling pathway for studying inhibitory synaptogenesis. We also demonstrated that intrahippocampal infusion or viral expression of the extracellular domain of Sema4D reduces seizure activity in vivo (Acker et al., 2018; Adel et al., 2023), suggesting that enhancing inhibition through synaptogenic signaling could represent a novel therapeutic avenue for disorders involving E/I imbalance, including epilepsy, autism spectrum disorders, and schizophrenia.

Semaphorins are a large family of secreted and transmembrane proteins (Kolodkin et al., 1993; Tamagnone et al., 1999). In addition to synaptogenesis (Paradis et al., 2007; Kuzirian et al., 2013; Acker et al., 2018; Frias et al., 2019; Adel et al., 2023), Semaphorins and their receptors (Plexins and Neuropilins) have been implicated in a variety of developmental processes including axon guidance, retinal lamination, neuronal migration, and vascular and heart morphogenesis (Joo et al., 2013; Duan et al., 2014; Koropouli and Kolodkin, 2014; Uesaka et al., 2014). Sema4D is a transmembrane protein that can be cleaved from the neuronal membrane, allowing its extracellular domain to signal either in cis or in trans to Plexin-B1 receptors (Raissi et al., 2013). Plexin-B1, which binds Sema4D with high affinity through its extracellular Sema domain (Tamagnone et al., 1999; Love et al., 2003; Alto and Terman, 2017), is required in both the presynaptic and postsynaptic neuron for rapid Sema4D-induced synapse formation (McDermott et al., 2018; Adel et al., 2024), suggesting that Sema4D/Plexin-B1 signaling may synchronously coordinate pre- and postsynaptic changes.

Taking advantage of these properties, in this study, we reveal how Sema4D signaling coordinates the spatiotemporal dynamics of pre- and postsynaptic protein assemblies to drive synapse formation. To do so, we leverage the unique ability of Sema4D to drive GABAergic synapse formation with temporal precision combined with two-channel live imaging of fluorescently labeled GABAergic synaptic proteins. By exploiting Sema4D/Plexin-B1 signaling as a rapid, selective inducer of inhibitory synapse formation, we overcome the inherent asynchrony of synaptogenesis and directly dissect mechanisms of individual synapse formation events. These experiments illuminate the downstream cellular consequences of trans-synaptic signaling and the fundamental molecular logic by which nascent axodendritic contacts are transformed into functional inhibitory synapses.

## Materials and Methods

### Ethics statement

All animal procedures were approved by the Brandeis University Institutional Animal Care and Usage Committee, and all experiments were performed in accordance with relevant guidelines and regulations.

### Animals

Male GAD65-GFP transgenic mice (López-Bendito et al., 2004) were obtained courtesy of Dr. Adam Puche (University of Maryland School of Medicine) and maintained in our animal facility with *ad libitum* access to food and water on a 12 h day/night cycle. Heterozygous GAD65-GFP males were crossed to female B6CBAF1/J mice (Jackson Laboratories, #100011) to produce litters in which approximately half of pups express one copy of the GAD65-GFP transgene. As this line expresses bright visible green fluorescence throughout the brain and spinal cord, GAD65-GFP pups were identified using a handheld 488 nm laser and an orange emission filter. For rat hippocampal cultures, pregnant female Sprague Dawley rats were obtained from Charles River Laboratories when litters were approximately Embryonic Day (E)14 and kept in our facility until dissection.

### Primary mouse hippocampal cultures

For GAD65-GFP and Halo-Gephyrin live-imaging experiments, primary rat astrocytes were plated onto 35 mm Petri dishes with glass-bottom 14 mm microwells (MatTek #P35G-1.5-14-C) that had been coated overnight at 37°C with poly-d-lysine (20 µg/ml) and laminin (3.4 µg/ml). Before plating glia, coverslips were washed three times with sterile Ultrapure water and once with DMEM (Invitrogen #10313039). Glia were plated in DMEM with FBS (GenClone #25-550) and grown in a 37°C incubator with 5% CO_2_ until confluent. When glia formed a confluent feeder layer, AraC (Sigma-Aldrich #C1768) was added at a final concentration of 5 µM in the dish to prevent further division. At Postnatal Day (P)0–1, GAD65-GFP mouse pups were identified as described above and rapidly killed by decapitation. Hippocampi were harvested from GAD65-GFP pups of both sexes, dissociated with papain (20 units/ml) for 8 min, and gently resuspended in Neurobasal medium (Invitrogen #21103049) with B27 supplement (Invitrogen #17504044; NB/B27) before plating atop glia within microwells at a density of 180 k cells/well. After 4–24 h when neurons were fully adhered, the plating medium was replaced by 1.5 ml NB/B27 with 5 µM AraC; the culture media were not changed thereafter.

For GFP-Gephyrin and Halo-GABA_A_Rγ2 overexpression live-imaging experiments, astrocytes were grown on 14 mm glass-bottom microwell and 35 mm Petri dishes as described above. Pregnant female Sprague Dawley rats were killed at E18 by CO_2_ asphyxiation, pups were rapidly removed and decapitated, and heads were kept in ice-cold dissociation media prior to dissection. Hippocampi were then dissected from pups of both sexes, dissociated, and resuspended in NB/B27 before plating atop astrocytes at a density of 120 k cells/well. After 4–24 h the plating medium was replaced by 1.5 ml NB/B27 and were not changed thereafter, except during transfection (see below).

### Infection/transfection and HaloTag labeling

For GAD65-GFP and Halo-Gephyrin live-imaging experiments, neurons were infected on 2 d in vitro (DIV) or DIV3 with AAV9.hSyn-HaloTag-Gephyrin virus (custom-produced by Duke Viral Vector Core) at a final concentration of 1 × 10^9^ GC/ml (∼8.33 × 10^3^ GC/neuron) in the dish. Culture media were not changed after addition of the virus. For labeling of HaloTag-expressing neurons, Janelia Fluor 646 HaloTag Ligand (Promega #GA1110) was suspended in DMSO according to manufacturer recommendations to create a 200 µM stock solution which was aliquoted and stored at −20°C for up to 1 year. To prepare the labeling solution, this stock was diluted 1:200 in NB/B27 to create a 1 µM 5× working stock. At DIV10–11, cultures were live labeled by aspirating all but 80 µl of growth media from each dish and adding 20 µl of 5× dye solution for a final concentration of 200 nM dye in the well. Final dilution was such that DMSO comprised no more than 0.1% of the total media volume during labeling. Cultures were incubated with ligand solution at 37°C for 15 min. After labeling, cells were briefly washed once with standard NB/B27 media which were then replaced with phenol red-free NB/B27 media prior to imaging. (Note: although excess unbound JF646 HaloTag ligand is reported to be minimally fluorescent, we observed greatly improved signal-to-noise after a brief wash.)

For GFP-Gephyrin and Halo-GABA_A_Rγ2 live-imaging experiments, DIV4 cultures were transfected with Lipofectamine 2000 using a protocol adapted from Marwick and Hardingham (2017). Lipofectamine 2000 reagent (3 µl/ng DNA) was diluted to a final volume of 33 µl per well using NB/B27; plasmid DNA (650 ng total split evenly between the two constructs) was separately diluted to a final volume of 33 µl per well with NB/B27. The L2000 solution was added to the DNA solution, pipetted 5–6×, and left to incubate at RT for 20 min. After incubation, the transfection mix was diluted to final volume of 125 µl per well using NB/B27. Working one dish at a time, growth media were aspirated from each dish and quickly replaced with 125 µl diluted transfection mix, and cells were incubated with transfection mix for 2–3 h at 37°C. Recovery media were then made by mixing 80% saved growth media with 20% fresh NB/B27, and transfection media were fully aspirated and replaced with 1.5 ml recovery media. The media were not changed again prior to labeling. At DIV10–11, Janelia Fluor labeling was performed as described above.

### Live imaging

Live images were obtained using an inverted Nikon AX-R Resonance Scanning Confocal with Ti2 body, Nikon Perfect Focus, a piezo Z controller, and a MRD71670 Plan Apochromat Lambda D 1.42 NA 60× oil objective. Culture dishes were placed into a humidified environmental enclosure maintained at 37°C with 5% CO_2_ at a constant flow rate of 0.2 L/min. Cells were allowed to habituate for at least 15 min prior to imaging. Single GAD65-GFP–positive cells were identified and a field of view where distal axons were clearly visible was chosen. Cultures were treated with 2 nM human IgG1-Fc control (R&D Systems #110-HG) or 2 nM recombinant Sema4D-Fc chimera (R&D Systems #7470-S4) by pipetting directly into the dish. Image acquisition was started immediately after adding the protein and setting the focal plane, typically within 1–2 min of adding each treatment. For experiments with GAD65-GFP and Halo-Gephyrin, we used 488 and 640 nm laser lines from a LUA-S4 laser unit at 2.5 and 3.0% power, respectively, and for experiments with GFP-Gephyrin and Halo-γ2, we used 488 and 640 nm lines with 5.2 and 4.0% power, respectively. The 12-bit images were acquired at a 2,048 × 2,048 resolution with a pixel density of 126.6 nm/px using the resonant scanner and 8× line averaging. A *Z*-stack of 5–7 Nyquist-sampled planes encompassing a total range of 1.5–2.1 µm was acquired at 10 s intervals (for GAD65-GFP only imaging experiments) or 15 s intervals (for dual GAD65-GFP/Halo-Gephyrin or GFP-Gephyrin/Halo-γ2 imaging experiments) for 1 h with optical focusing correction (Nikon Perfect Focus) to minimize drift in the *Z* direction.

### Image unwarping and registration

To eliminate the possibility that changes to protein cluster mobility were due to cell motility, changes to dendrite/axon morphology, or image drift in the *X*–*Y* plane, images were registered and unwarped prior to particle tracking analysis. Time-lapse images were first corrected for any stage *X*–*Y* drift during acquisition using the Linear Stack Alignment with SIFT plugin in ImageJ with the following parameters: initial Gaussian blur 1 px, steps per octave 8, image size 100–250 px, feature descriptor size 4, feature descriptor orientation bins 8, closest/next closest ratio 0.96, maximum alignment error 5 px, inlier ratio 0.05, and rigid transform.

Following linear alignment, images were next unwarped using the BigWarp plugin in ImageJ (Bogovic et al., 2016) to correct for nontranslational drift (movement of axons/dendrites, etc.). Briefly, time lapses were flattened into maximum intensity projections and then converted to a virtual *Z*-stack. The first frame of each image was duplicated repeatedly (once per timepoint) and converted to a virtual *Z*-stack to form a reference stack that was identical in size to the moving image stack. The moving image stack and reference stack were then imported into BigWarp viewer, and pairs of landmarks were manually chosen in the last plane of the moving image (corresponding to the last timepoint of the time lapse) and the reference stack. To ensure proper linear interpolation of the transformation grid across time, the moving image was split into 30-frame intervals, and landmarks in the moving image were linearly interpolated at the corresponding 30-frame intervals. The 30-frame subvideos were then individually aligned to the interpolated landmarks using a thin-plate splines transform, which uses a deformable grid to perform exact matching between the moving image and reference image landmarks. Transformations were applied using BigWarp Apply with the following parameters: thin-plate spline transformation; bounding type FACES; samples, 5; and linear interpolation. For each 30-frame subvideo, the last frame of the unwarped output served as the reference stack to which the next subvideo was aligned; this prevents compounding error over time. Unwarped 30-frame subvideos were finally stitched together and converted back to a time series for particle tracking analysis.

### Qualitative scoring of GAD65-GFP protein cluster behavior

Primary hippocampal cultures were generated from P0–1 GAD65-GFP mice as described above. DIV2 cultures were infected with a virus expressing HaloTag-Gephyrin under the synapsin promoter. Cultures were treated with 2 nM Sema4D-Fc or Fc control protein at DIV11 as previously described and imaged at 10 s intervals for 1 h. To qualitatively assess cellular processes that may be relevant to synapse formation, the following categories of behaviors were chosen based on empirical observation: trafficking, nascent branching, local cluster mobility, splitting, merging, complex split/merge events, active growth cones, stable branch formation, and stable branch removal. Complete descriptions of these behaviors are found in Extended Data Table 1-1. An experimenter blinded to condition manually traced 10 axons (including branches) per cell using the freehand line tool in ImageJ. Axons were selected only if the majority of the process remained in focus across all time points. Behaviors were manually counted by an experimenter blinded to condition and the frequency of each behavior was normalized to the total length of each axon.

### Particle tracking for live images

For all analyses of protein cluster mobility and real-time colocalization analysis, we utilized the surface tracking feature in Imaris for Neuroscientists v10.2.0 (Oxford Instruments). To facilitate accurate tracking of low-intensity protein clusters, live images were denoised using onboard Nikon Denoise.ai in Nikon Elements prior to generating max intensity projections. Max intensity projections were then imported into Imaris for tracking with both the denoised and raw fluorescence channels. Surfaces were created in the GAD65-GFP, Halo-Gephyrin, GFP-Gephyrin, and/or Halo-γ2 channels using the machine learning (ML) segmentation feature in Imaris with the denoised channel; we determined empirically that this led to more consistent identification of protein clusters than standard background subtraction and thresholding, particularly for bright GAD65-GFP clusters along axons. We next tracked surfaces across frames using the following parameters: autoregressive motion tracking, max frame-to-frame distance 2 µm, and max frame gap 8. Tracks were automatically filtered out if their duration was <60 s and were manually removed if they showed spurious linkages between adjacent neurites or if they were located on a neurite that did not appear to be an axon (for GAD65-GFP) or a dendrite (for gephyrin or Halo-γ2). For most analyses, only protein clusters that could be tracked for the duration of the live-imaging session were considered for further analysis.

### Analysis of protein cluster stability

Following automated particle tracking in Imaris, we quantified the stability of each tracked puncta as the proportion of frames within its tracked interval in which the puncta was directly detected by the ML segmentation model, following the approach of Frias et al. (2019). For a given track spanning frames *f*_0_ to *f_n_*, where each frame *f_i_* represents a frame in which the puncta was positively segmented, stability was calculated as $\mathrm{stability}=((|f_{i}|)/(f_{n}\;-\;f_{0}))$, where $\;|\;f_{i}|$ is the total number of frames in which the puncta was positively segmented and tracked by the tracking algorithm and *f_n_* − *f*_0_ is the total number of frames spanned by the track. Frames within the tracked interval that lacked a direct detection but were bridged by the autoregressive tracking algorithm (max gap, eight frames) were not counted toward $\left|\;f_{i}\right|$. Thus, stability is mathematically independent of track duration: a puncta tracked over a short interval but detected in every frame yields the same stability value (1) as a puncta tracked across the full imaging session without gaps. Instead, stability represents the degree to which a given protein assembly produces a consistent, above-threshold fluorescence signal during its observable interval. Permitting a maximum gap of eight frames during tracking was essential for this measurement, as it allowed inclusion of puncta with frequent dropouts (consistent with unstable or immature protein assemblies) rather than excluding them as broken tracks.

### Analysis of protein cluster mobility

#### Surface and track features

All analyses were performed in MATLAB R2025a (MathWorks) and visualized in GraphPad Prism 10.5.0. Surface and track statistics for each channel were exported from Imaris and fed into a custom MATLAB analysis pipeline for data workup. Surface features that were exported for analysis included surface area, acceleration, displacement delta length, intensity in each channel, position, overlapped area ratio, speed, and nearest neighbor distance to other surfaces. Track features that were exported for analysis included track duration, length, and number of surfaces. Other track features were calculated within our analysis pipeline, such as raw change in fluorescence (*F*_end_ − *F*_0_), fold change fluorescence (*F*_end_ / *F*_0_), peak fold change fluorescence (*F*_max_ / *F*_0_), and Euclidean distance (net displacement between selected timepoints).

#### Fluorescence intensity analysis

All analysis of fluorescence intensity was performed using the raw fluorescence (i.e., nondenoised) channel. For analysis of protein cluster fluorescence intensity over time, a photobleach correction was applied by fitting a one-step exponential decay model to the average particle intensity in the control condition using $F(t)=A\cdot e^{-kt}+C$, where *A* is the amplitude, *k* is the decay rate constant, *t* is time in minutes, and *C* is the baseline offset. To correct photobleaching, the raw fluorescence was transformed using $\;F_{\mathrm{corrected}}(t_{n})=(F_{\mathrm{measured}}(t_{n})\cdot [F(t_{0})/F(t_{n})]\;)$. We then measured the average and total fluorescence intensity within the outline of each individual puncta for each timepoint. For figures measuring the change in normalized fluorescence intensity over time, fluorescence intensity was normalized to the baseline mean of the first 3 min (12 frames) for all puncta within each treatment condition.

#### New colocalization event analysis

New colocalization events were defined as instances in which a GAD65-GFP bouton that was not colocalized with a gephyrin puncta (or vice versa) in the previous frame became colocalized, which we determined by comparing the nearest neighbor distance to a Halo-Gephyrin puncta between frames. To avoid counting transient crossings, both puncta were required to remain colocalized in at least 95% of frames over the next 10 min following the initial colocalization event (colocalization events happening in the final 10 min of the imaging session were not analyzed). To determine whether the puncta in the opposite channel was newly formed (new puncta) or previously present (existing puncta), we identified puncta in the opposite channel for which the center of mass was located within 1 µm of the colocalization site at the colocalization timepoint and used this criterion to determine the unique track ID of the opposite-channel puncta. If the opposite-channel puncta was present at least 10 min before the colocalization event, it was considered an existing puncta; otherwise it was classified as a new puncta. After classifying new colocalization events, we then quantified average protein cluster velocity, fluorescence intensity, displacement from origin, and distance from the colocalization site in (1) the entire window before the new colocalization event and (2) the 10 min periods before and after the new colocalization event.

### Experimental design and statistical analysis

Statistical analyses for all figure panels except time series data were performed using GraphPad Prism 10.5.0. For each experiment, specific statistical tests are described in the figure legends. Data were tested for normality before appropriate statistical tests were applied. Where indicated, outliers were identified by the ROUT method with *Q* = 1% and removed from the dataset.

For time series data, to assess whether Sema4D treatment interacts with GAD65-GFP bouton features to regulate gephyrin accumulation in GAD65-GFP boutons, we fit binned time series data to linear mixed-effects models (LME) using MATLAB (fitlme), which was chosen to account for the effects of cell-to-cell variability on track measurements. Due to the large number of timepoints sampled in each image, timepoints were binned to avoid artificially inflating statistical power, with bin size determined by calculating autocorrelation at a range of bin sizes for the mean of the control condition across timepoints using MATLAB autocorr function. Bin size was fixed as the smallest round-number bin size at which the autocorrelation function dropped below 0.3, thus ensuring semi-independence of timepoints for the purposes of LME model fitting. Models tested the effects of treatment condition, time, GAD65-GFP bouton displacement length, area, and/or intensity and all two-way and three-way interactions on either the nearest distance between GAD65 and gephyrin puncta or mean fluorescence intensity of gephyrin within GAD65-positive regions. Random intercepts were used to account for variation between images (within-experiment variability) and between individual tracks within images (within-puncta variability over time). Model fit was assessed by inspecting residuals, random effects distributions, and summary fit indices (AIC, BIC, LogLikelihood). Effect estimates are reported as changes in the dependent variable per unit change in the predictor. Time-dependent effects are interpreted as rate changes per minute. For main effects and interactions with *p* < 0.05, follow-up analysis was conducted using subsets of puncta according to the variable of interest (e.g., a significant interaction between time, treatment, and puncta size was further analyzed by grouping puncta by quintiles according to size and plotting mean gephyrin fluorescence over time in each subset.) For experiments involving GFP-Gephyrin and Halo-γ2, statistical analysis of live-imaging parameters was performed identically to the above.

### Code availability

All ImageJ and Python scripts used for landmark interpolation, unwarping, and stitching are publicly accessible via GitHub at zpranske/Bigwarp_Analysis. All MATLAB scripts and functions used for statistical analysis of particle tracking data are publicly accessible via GitHub at zpranske/LiveImaging_Analysis_Imaris.

## Results

### Sema4D treatment differentially affects the stability and mobility of pre- and postsynaptic protein clusters

A long-standing question in synapse formation is whether synaptogenic signals act by stabilizing existing, dynamic protein assemblies at nascent contact sites or by driving the formation of entirely new ones. To distinguish between these possibilities, we first characterized the baseline dynamic behaviors of GABAergic presynaptic boutons and asked how Sema4D treatment alters them. For the purposes of this study, a synapse is operationally defined as stable colocalization of pre- and postsynaptic protein clusters (see Materials and Methods for detailed criteria); a nascent synapse refers to a pre- or postsynaptic protein cluster that has not yet colocalized with a synaptic partner, and a nascent synapse becomes a newly formed synapse at the moment of colocalization.

**Figure 1.**
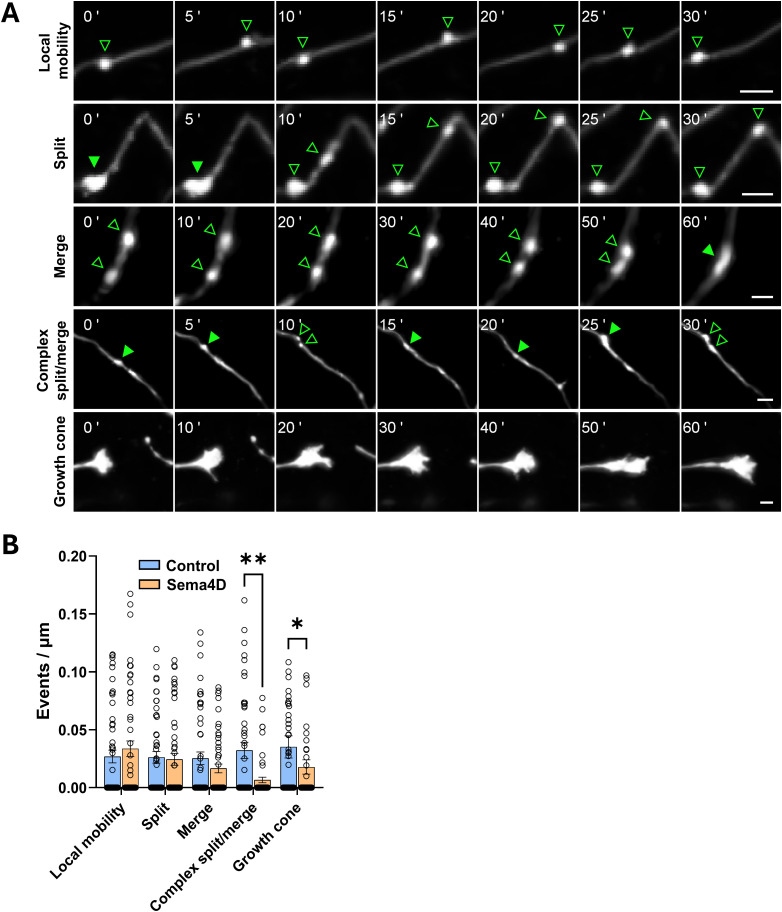
Sema4D alters dynamic behaviors of GAD65-GFP boutons. ***A***, Representative images of stretches of axon from DIV11 GAD65-GFP mouse hippocampal neurons showing dynamic behaviors of interest. Top to bottom, Local mobility (repeated movement of a single cluster along the axon), split, merge, complex split/merge, and growth cone events. Note: timescales differ between events; time points are relative to the start of the montage. Example images include cells from either treatment condition; some montages show different axonal regions from the same neuron. Hollow arrows, single puncta; solid arrows, merged puncta. Scale bars, 2 µm. ***B***, Frequency of local mobility, split, merge, complex split/merge, and growth cones observed in control versus Sema4D-treated cultures. Dots correspond to individual axons; *n* = 50–55 axons per condition from 11 cells (Fc) or 13 cells (Sema4D). *p < 0.05; **p < 0.01; Mann–Whitney *U* test. Refer to Extended Data Figure 1-1 for validation of GAD65 immunopositivity and Extended Data Table 1-1 for detailed descriptions of qualitative scoring parameters.

**Figure 2.**
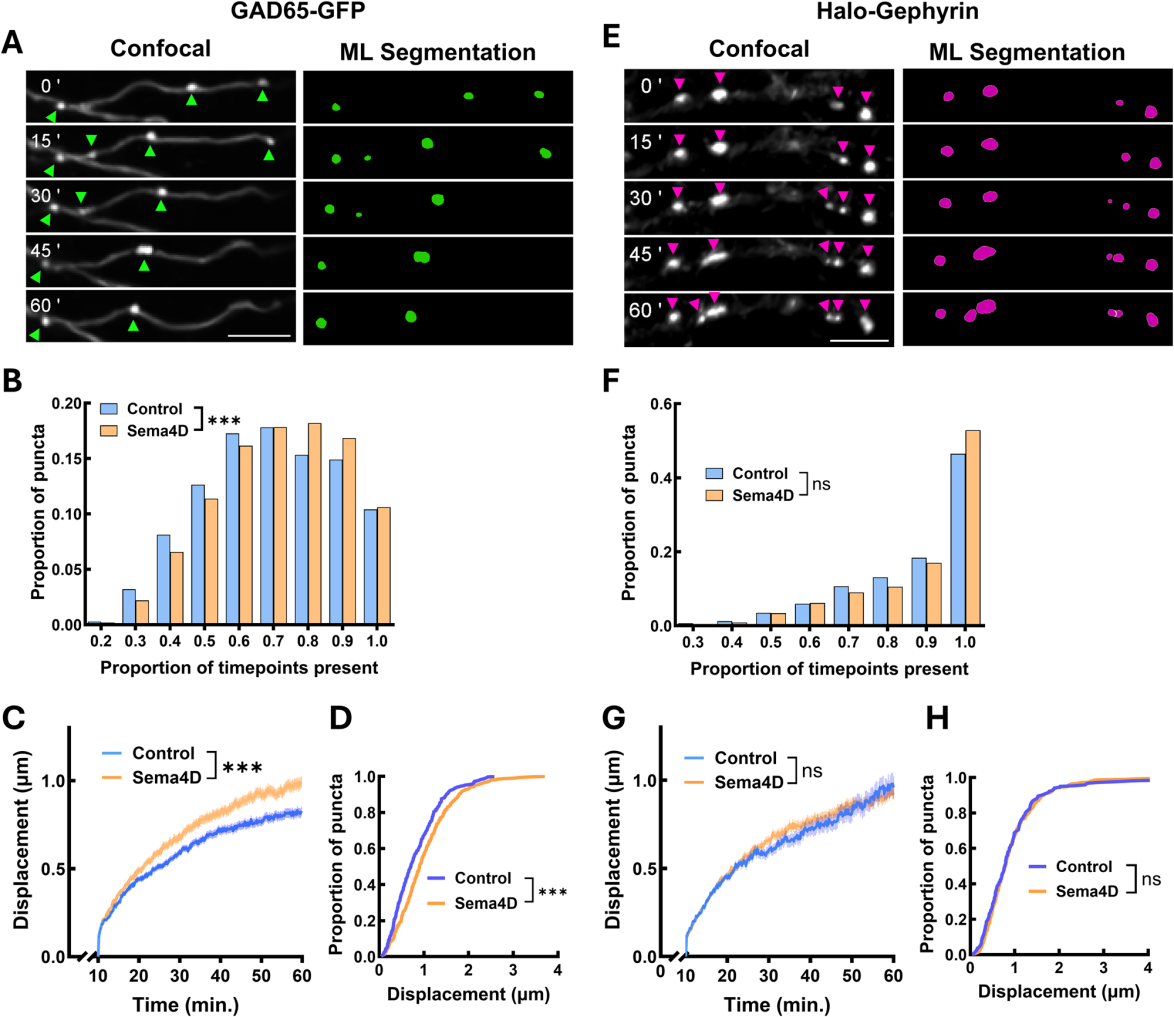
Sema4D treatment increases both the mobility and stability of presynaptic GAD65-GFP boutons without affecting postsynaptic gephyrin scaffold mobility. ***A***, Left, Example stretch of axon from cultured hippocampal GAD65-GFP neurons. GAD65-GFP boutons appear as bright spots marked by arrows. Right, ML reconstruction of GAD65-GFP boutons in Imaris at the same timepoints. Scale bar, 5 µm. ***B***, GAD65-GFP bouton stability (proportion of frames in which a puncta was tracked). *n* = 7,427 puncta (Fc), 10,213 puncta (Sema4D). The distributions of GAD65-GFP boutons are significantly different between treatment conditions (*χ*^2^ test; *χ*^2^_(8)_ = 72.66; ***p < 0.001). ***C***, GAD65-GFP bouton mobility (mean displacement from origin over the imaging session) is increased beginning within 20 min of Sema4D treatment (binned LME, time × treatment interaction, *F*_(1,3651)_ = 11.578; *p* < 0.001). *n* = 384 puncta from 10 cells (Fc); *n* = 347 puncta from 12 cells (Sema4D). Note: analysis begins at *t* = 10 min and ends at *t* = 60 min. ***D***, Cumulative frequency histogram of GAD65-GFP bouton displacement at 60 min (***p < 0.001; Kolmogorov–Smirnov test). *n* = 384 puncta from 10 cells (Fc); *n* = 347 puncta from 12 cells (Sema4D). ***E***, Representative stretch of dendrite showing Halo-Gephyrin expression along dendrites in cultures from GAD65-GFP neurons. Halo-Gephyrin scaffolds appear as bright spots marked by arrows. Right, ML reconstruction of Halo-Gephyrin scaffolds in Imaris at the same timepoints. Scale bar, 5 µm. ***F***, Halo-Gephyrin scaffold stability (proportion of frames in which a puncta was tracked). *n* = 742 puncta (Fc), 1,026 puncta (Sema4D). The distributions of Halo-Gephyrin scaffolds do not significantly differ between treatment conditions (*χ*^2^ test; *χ*^2^_(7)_ = 10.63; *p* = 0.1557). ***G***, Halo-Gephyrin scaffold mobility (mean displacement from origin over the imaging session) is not affected by Sema4D treatment (binned LME, time × treatment interaction, *F*_(1,7279)_ = 1.0464; *p* = 0.2954). *n* = 220 puncta from eight cells (Fc); *n* = 393 puncta from eight cells (Sema4D). Note: analysis begins at *t* = 10 min and ends at *t* = 60 min. ***H***, Cumulative frequency histogram of Halo-Gephyrin scaffold displacement at 60 min. There was no effect of Sema4D treatment on the overall distribution of Halo-Gephyrin displacement (*p* = 0.7637). *n* = 220 puncta from eight cells (Fc); *n* = 393 puncta from eight cells (Sema4D). Refer to Extended Data Figure 2-1 for quantification of GABAergic synapse density in cells expressing Halo-Gephyrin.

We generated primary hippocampal neuronal cultures from P0–1 GAD65-GFP mice, in which a subset of interneurons express a transgenic GAD65-GFP fusion protein (López-Bendito et al., 2004). In these animals, axons are identified by diffuse, low-intensity GFP expression along neuronal processes, while presynaptic boutons are distinguishable as bright GFP-positive puncta (Figs. 1*A*, 2*A*; Extended Data Fig. 1-1). Previous characterization of this mouse line demonstrated that GAD65-GFP puncta represent genuine presynaptic GABAergic boutons by colocalization with the GABAergic, presynaptic protein VGAT, and the postsynaptic marker gephyrin (Wierenga et al., 2008; Schuemann et al., 2013; Frias et al., 2019). We independently confirmed that these GFP-labeled boutons contain GAD65 by immunostaining with a GAD65-specific antibody (Extended Data Fig. 1-1).

10.1523/ENEURO.0140-26.2026.f1-1 Figure 1-1 **Boutons marked by GAD65-GFP are immunopositive for GAD65 protein.** GAD65-GFP labeled boutons reliably colocalize with GAD65 antibody staining in distal axons of primary cultured DIV11 hippocampal neurons from GAD65-GFP mice. Green arrows = GAD65-GFP; magenta arrows = anti-GAD65. The majority of GAD65-GFP boutons in GFP-positive cells are marked by anti-GAD65 antibody (white arrows = colocalized). Scale bars = 10 µm. Download Figure 1-1, TIF file.

10.1523/ENEURO.0140-26.2026.t1-1 Table 1-1 Descriptions of qualitative scoring parameters. Download Table 1-1, TIF file.

10.1523/ENEURO.0140-26.2026.f2-1 Figure 2-1 **Viral expression of Halo-Gephyrin does not increase GABAergic synapse density.** (A) Virally-expressed Halo-Gephyrin in cultured DIV11 rat neurons colocalizes with anti-GAD65 antibody. Magenta = Halo-Gephyrin, green = anti-GAD65 antibody, white = colocalized. Scale bar = 10 µm. (B) Sample stretches of dendrite from DIV11 rat neurons expressing GFP (green) with or without Halo-Gephyrin. Scale bar = 5 µm. (C) Viral Halo-Gephyrin expression marginally increases gephyrin puncta density compared to no-virus control neurons (unpaired t-test, p = 0.0503). n = 55 neurons from 2 replicates per condition. (D) Viral Halo-Gephyrin expression does not affect GAD65 puncta density compared to no-virus control neurons (unpaired t-test, p = 0.2753). n = 55 neurons from 2 replicates per condition. (E) Viral Halo-Gephyrin expression does not affect colocalized GAD65/gephyrin synapse density compared to no-virus control neurons (unpaired t-test, p = 0.1719). n = 55 neurons from 2 replicates per condition. Download Figure 2-1, TIF file.

Dissociated GAD65-GFP hippocampal neurons were plated atop an astrocyte feeder layer and cultured for DIV10. At DIV10–11, we treated cultures acutely with 2 nM control protein (Fc domain of human IgG_1_ recombinant protein) or 2 nM Sema4D-Fc (recombinant protein containing the soluble extracellular domain of Sema4D fused to Fc). Immediately following addition of the recombinant proteins, we acquired images of GAD65-GFP–positive neurons using a Nikon AX-R resonant scanning confocal microscope to obtain *z*-stacks of five Nyquist-sampled planes at 10 s intervals for 1 h. We chose this experimental time course to focus on the synaptogenic window between initiation of Sema4D signaling and emergence of synaptic functionality in order to gain a cell biological view of the molecular and structural events that give rise to functional inhibitory synapses.

GAD65-GFP boutons and their associated axons exhibited a range of dynamic behaviors including splitting events, merging events, rapid local protein cluster mobility, complex split/merge events, nascent axonal branching, and active growth cones (Fig. 1*A*). We manually characterized the behaviors of GAD65-GFP–labeled boutons by identifying and quantifying these events of interest (Fig. 1*B*; for complete definitions, see Extended Data Table 1-1). Sema4D treatment significantly decreased the frequency of complex split/merge events, defined as multiple or repeated splitting and merging behavior of one or more GAD65-GFP boutons, compared with control treatment (Fig. 1*B*). Sema4D treatment also decreased the number of active axonal growth cones observed, consistent with its canonical role in growth cone collapse (Oinuma et al., 2004; Ito et al., 2006), thus confirming that Sema4D-Fc protein is active in our cultures. In contrast, the rates of single split, single merge, and locally restricted mobility events (boutons moving within a small radius without splitting or merging) of GAD65-GFP boutons, as well as the rate of nascent axonal branch formation, were unaffected. This suggests that Sema4D signaling has a role in stabilizing a specific subset of immature, mobile presynaptic protein assemblies that undergo repeated split/merge events.

To track the mobility of protein puncta quantitatively, we identified puncta at each timepoint using a custom-trained ML segmentation model in the Imaris for Tracking suite (Oxford Instruments) and empirically determined that this rigorous approach produced the highest fidelity puncta identification over time (Fig. 2*A*,*E*). We then performed automated particle tracking using Imaris and a custom analysis suite in MATLAB. We first quantified presynaptic GAD65-GFP bouton stability by calculating the proportion of imaging frames in which each bouton was positively identified by the ML segmentation model (excluding frame gaps bridged by the tracking algorithm) during the lifespan of that specific bouton (see Materials and Methods). Stability thus quantifies how consistent a bouton's fluorescence was during the interval in which it was tracked, reflecting the stability of the presynaptic structure. Sema4D treatment shifted the distribution of bouton stability rightward (Fig. 2*B*), indicating that Sema4D treatment stabilizes existing GAD65-GFP boutons.

Whether GABAergic synapse formation is initiated from the presynaptic or postsynaptic side or both is a fundamental open question, with evidence from excitatory synapse studies suggesting that the presynaptic compartment leads the process (Ahmari et al., 2000; Friedman et al., 2000; Roos and Kelly, 2000; Bresler et al., 2001, 2004). To characterize the mobility of individual GAD65-GFP boutons in response to Sema4D, we focused on GAD65-GFP boutons that were consistently tracked between *t* = 10 min and *t* = 60 min of the imaging session. Beginning the analysis at *t* = 10 min was necessary due to an initial, transient rise in mean displacement distance for all GAD65-GFP boutons within this time window in both treatment conditions, presumably due to physical perturbation of axons by addition of the protein to the culture media or imaging onset. We first characterized overall GAD65-GFP bouton mobility by tracking displacement of every identified GAD65-GFP bouton in the field of view starting at *t* = 10 min. We found that Sema4D treatment led to an overall increase in mean GAD65-GFP bouton mobility beginning ∼20 min after addition of Sema4D protein, as shown by a significant increase in mean displacement in Sema4D-treated cultures compared with control from ∼20–60 min (Fig. 2*C*). We next compared the overall distributions of GAD65-GFP bouton mobility across the population after 60 min of Sema4D or control treatment. Across both treatment conditions, the majority (∼90%) of GAD65-GFP boutons were relatively immobile, displacing <2 µm from their initial position, consistent with localization at stable presynaptic sites (Fig. 2*D*). However, we observed that overall mean displacement was significantly increased in Sema4D-treated cultures at this timepoint (Fig. 2*D*). The shape of the cumulative distribution was similar between treatment conditions, suggesting that Sema4D induces a population-wide enhancement to presynaptic protein cluster mobility rather than creating a distinct subpopulation of highly mobile boutons.

Overall, the results from these experiments (Figs. 1, 2*A*–*D*) indicate that a subset of GAD65-GFP protein clusters are mobile and display dynamic behaviors during the 1 h imaging session. Sema4D-dependent changes to presynaptic GAD65-GFP bouton mobility are time-dependent, with an overall increase in mean bouton mobility beginning ∼20 min after Sema4D addition. The observation that increased mobility is accompanied by increased bouton stability and a decrease in the frequency of complex split/merge events suggests that Sema4D increases exploratory behavior of presynaptic boutons which may facilitate recruitment of a postsynaptic specialization, leading to stabilization.

We next asked whether Sema4D similarly affected the dynamics of the postsynaptic specialization. We generated primary hippocampal cultures from P0–1 GAD65-GFP mice as described above and infected DIV2 neurons with an AAV9 virus expressing HaloTag-Gephyrin under the control of the pan-neuronal hSyn promoter (Halo-Gephyrin) to allow for visualization of postsynaptic scaffold assemblies (Fig. 2*E*; Extended Data Fig. 2-1). We confirmed that virally expressed Halo-Gephyrin colocalizes with endogenous gephyrin (Extended Data Fig. 2-1*B*) and localizes to synapses as revealed by coimmunostaining with a GAD65-specific antibody (Extended Data Fig. 2-1*A*,*B*). While gephyrin puncta density is marginally increased in these cultures (Extended Data Fig. 2-1*C*), GABAergic synapse density was unaffected (Extended Data Fig. 2-1*E*) suggesting that virally expressed Halo-Gephyrin does not by itself drive synapse formation. At DIV10–11, we labeled cultures with Janelia Fluor 646 (JF646) HaloTag ligand to visualize Halo-Gephyrin and then treated with 2 nM Sema4D or Fc control protein and acquired images at 15 s intervals for up to 1 h using a resonant scanning confocal microscope. As before, we utilized a custom-trained ML segmentation network, automated particle tracking in Imaris, and custom MATLAB-based analysis software to analyze the mobility of Halo-Gephyrin puncta (Fig. 2*E*).

In contrast to GAD65-GFP boutons, we observed no effect of Sema4D treatment on Halo-Gephyrin scaffold stability over time (Fig. 2*F*). Similar to GAD65-GFP, 90–95% of Halo-Gephyrin scaffolds were relatively immobile, displacing <2 µm from their starting location (Fig. 2*G*). We did not observe a Sema4D-dependent effect on mean Halo-Gephyrin displacement (Fig. 2*G*) or the distribution of Halo-Gephyrin scaffold mobility at the population level (Fig. 2*H*). Thus, we concluded that Sema4D does not affect the overall mobility of gephyrin-positive postsynaptic scaffolds within the first hour. Taken together these data suggest that Sema4D promotes GABAergic synapse formation primarily by preferentially altering the behavior of the presynaptic bouton.

### Sema4D treatment drives gephyrin localization to postsynaptic sites adjacent to GAD65-GFP–labeled boutons

**Figure 3.**
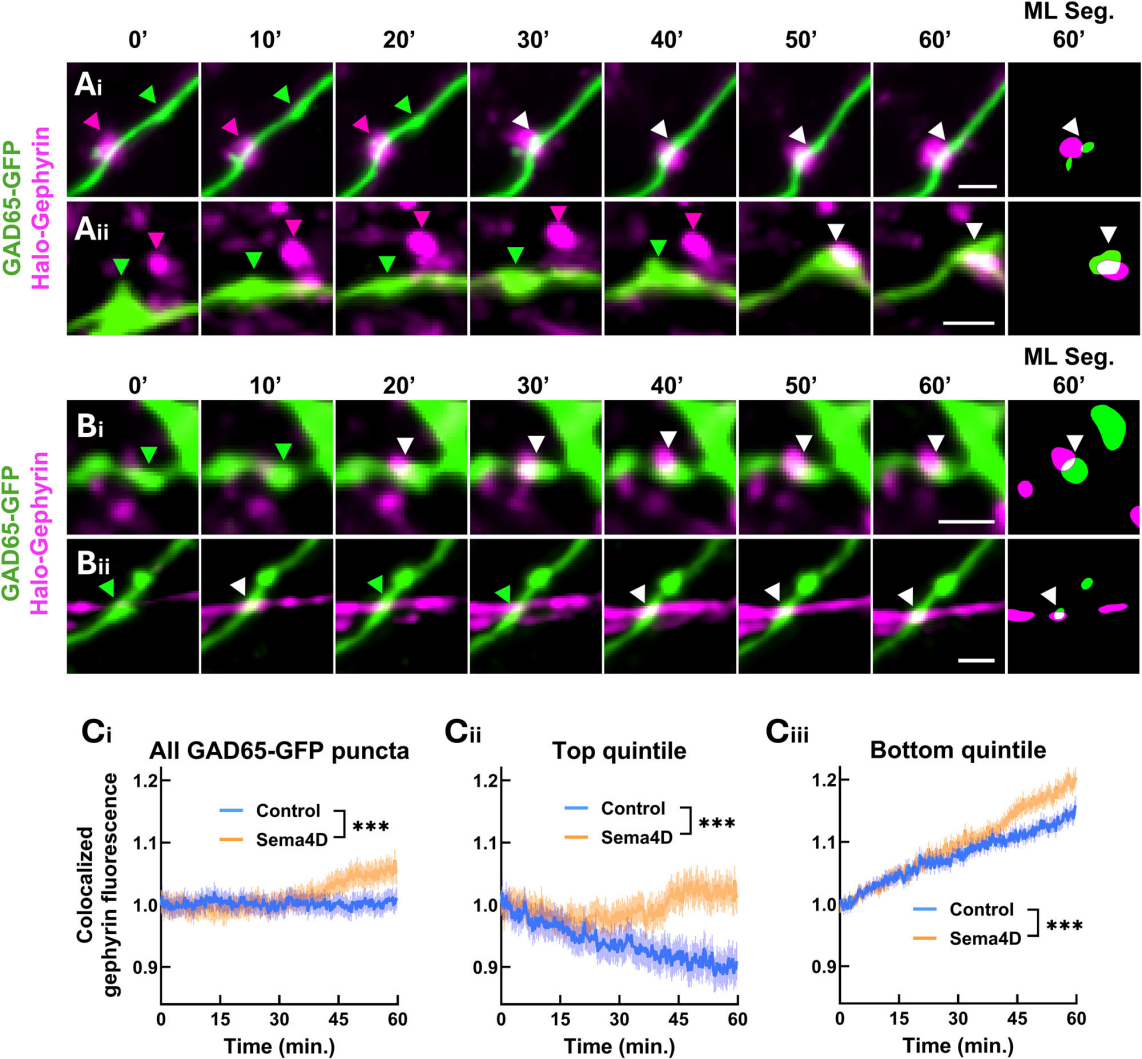
Acute Sema4D treatment recruits gephyrin to sites marked by existing GAD65-GFP boutons. ***A***, Montages showing putative new synapse formation events in which existing mobile GAD65-GFP boutons (green arrows) localize to existing Halo-Gephyrin scaffolds (magenta arrows) and remain colocalized. White arrows, colocalized puncta. Scale bars, 2 µm. ***B***, Montages showing putative new synapse formation events in which a newly formed Halo-Gephyrin scaffold emerges and colocalizes with an existing GAD65-GFP bouton (green arrows). White arrows, colocalized puncta. Scale bars, 2 µm. ***C***, ***i***, Gephyrin fluorescence colocalized with GAD65-GFP boutons is increased in Sema4D condition compared with control (binned LME, time × treatment interaction, *F*_(1,24140)_ = 13.656; ***p < 0.001). *n* ≥ 765 puncta per timepoint (control), 1,000 puncta (Sema4D). ***ii***, Sema4D prevents the loss of gephyrin from GAD65-GFP boutons in the top quintile of baseline gephyrin signal (interaction, *F*_(1,4820) _= 17.75; ***p < 0.001). *n* ≥ 151 puncta per timepoint (control), 201 puncta (Sema4D). ***iii***, Sema4D treatment increases recruitment of gephyrin to boutons in the bottom quintile of baseline gephyrin signal compared with control treatment (interaction, *F*_(1,4820)_ = 17.637; ***p < 0.001). *n* ≥ 150 puncta per timepoint (control), 197 puncta (Sema4D). Error bars indicate SEM. Data are normalized within treatment condition to the mean of the first 3 min.

Next, we sought to determine the time course over which pre- and postsynaptic markers of GABAergic synapses become spatially associated in response to Sema4D. While our previous immunostaining studies of fixed cells established that Sema4D signaling increases the density of inhibitory synapses (Paradis et al., 2007; Kuzirian et al., 2013; Raissi et al., 2013), they could not reveal the temporal dynamics by which pre- and postsynaptic components become colocalized. Because our strategy labels a subset of GABAergic boutons with GAD65-GFP while marking almost all postsynaptic sites with Halo-Gephyrin (Extended Data Fig. 2-1), we focused on stably tracked GAD65-GFP boutons and asked whether Sema4D treatment promotes increased accumulation of Halo-Gephyrin signal at adjacent postsynaptic sites. We measured the mean, baseline-normalized fluorescence intensity of the Halo-Gephyrin channel within the area defined by each GAD65-GFP bouton (Fig. 3*A*,*B*); we refer to this readout hereafter as “colocalized gephyrin fluorescence.” We found that Sema4D treatment significantly increased mean colocalized gephyrin fluorescence beginning at ∼40 min. (Fig. 3*C*i), consistent with our previous results in fixed cells.

To assess whether Sema4D-dependent recruitment of gephyrin to GAD65-GFP boutons varied as a function of the initial degree of postsynaptic association, we analyzed subsets of GAD65-GFP boutons grouped by their baseline levels of colocalized gephyrin fluorescence. The top quintile, characterized by greater colocalized gephyrin fluorescence at *t* = 0 min, showed a gradual decrease in colocalized gephyrin fluorescence over time in the control condition; Sema4D treatment prevented the loss of gephyrin fluorescence from this subset (Fig. 3*Cii*). In contrast, the bottom quintile of GAD65-GFP boutons, characterized by little-to-no colocalized gephyrin fluorescence initially, showed gradually increasing colocalized gephyrin fluorescence over time in control and Sema4D-treated neurons. Sema4D treatment led to further increased colocalized gephyrin fluorescence beyond the level seen in control neurons, suggesting additional recruitment of gephyrin to this subset of GAD65-GFP boutons in response to Sema4D treatment (Fig. 3*Ciii*). Overall, these data suggest that Sema4D acts to (1) stabilize and prevent the loss of gephyrin from sites of colocalization with presynaptic boutons and (2) promote localization of gephyrin at presynaptic boutons that were previously lacking gephyrin.

### The spatiotemporal dynamics of synaptic protein clusters during single putative synapse formation events support a presynapse-first model of Sema4D-induced synapse assembly

To date, the spatiotemporal dynamics of single synapse formation events are poorly understood. Outstanding questions include when synaptic protein clusters become colocalized, in which synaptic compartment were proteins mobilized first? When is each participating protein cluster mobile relative to the moment it becomes localized to the new synapse? A key advantage of using Sema4D to synchronize inhibitory synapse assembly is that many individual putative synapse formation events can be observed within 1 h allowing us to dissect the precise spatiotemporal dynamics of single synapse formation events.

To begin, we defined new colocalization events between GAD65-GFP and gephyrin as events in which a noncolocalized GAD65-GFP bouton became colocalized with Halo-Gephyrin over the course of our imaging session. To eliminate the possibility of detecting transient crossings between mobile protein puncta, new colocalization events were considered genuine only if they occurred between a pair of GAD65-GFP and Halo-Gephyrin puncta that were not colocalized in the previous 10 min and then remained stably colocalized for at least 10 min. Because GAD65-GFP labels a sparse population of presynaptic boutons, we considered that some gephyrin puncta involved in new colocalization events may have been previously apposed to an unlabeled bouton. However, because the ultimate result of Sema4D treatment is a net increase in synapse density (Kuzirian et al., 2013; Raissi et al., 2013) and redistribution of material from existing synapses to new synaptic sites is a plausible mechanism of synapse assembly (see Discussion), the prior association history of individual gephyrin puncta should not affect the interpretation of this analysis.

**Figure 4.**
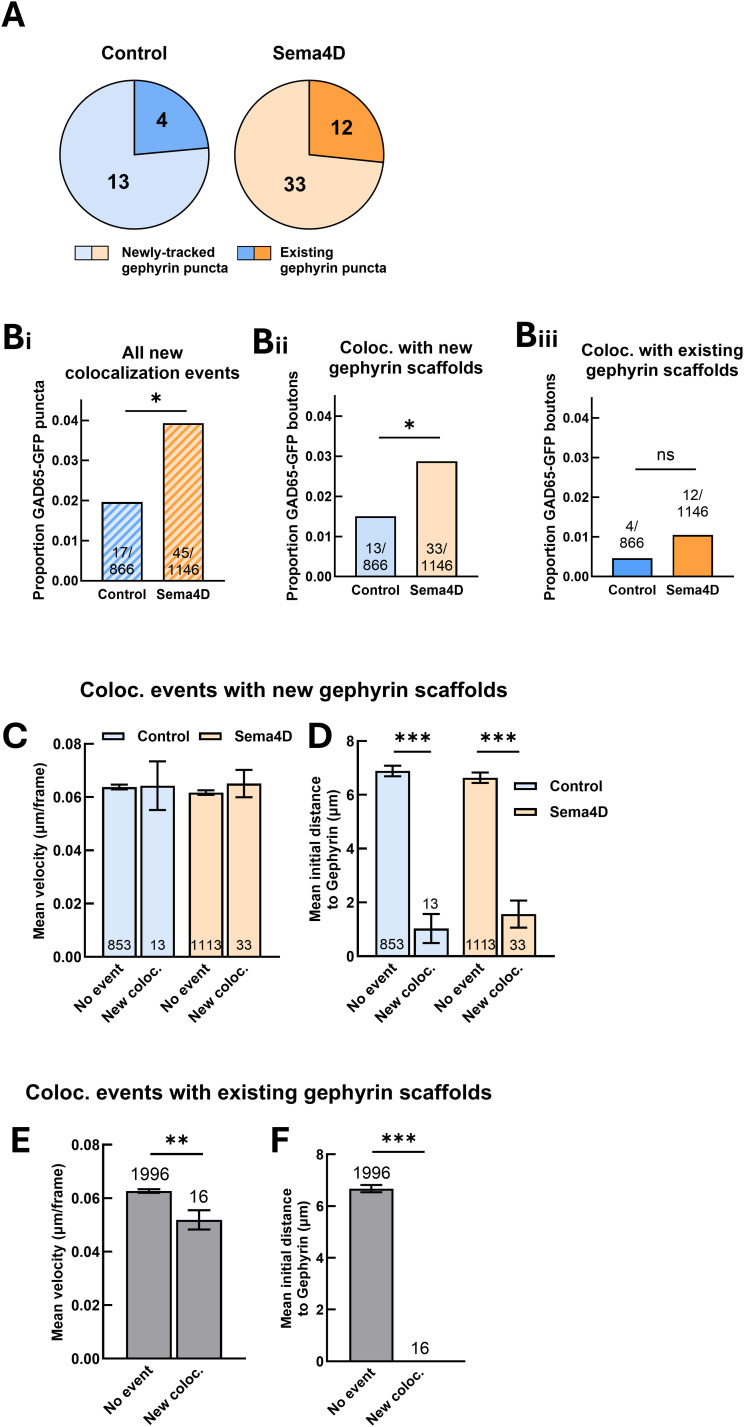
Sema4D-dependent GAD65–gephyrin colocalization occurs via assembly of new gephyrin scaffolds. ***A***, Number of GAD65-GFP boutons that colocalized with an existing Halo-Gephyrin scaffold versus a newly formed Halo-Gephyrin scaffold. ***B***, ***i***, Overall frequency of all new colocalization events. Sema4D treatment significantly increased the frequency of new colocalization events compared with control (*p < 0.05). ***ii*** Sema4D treatment significantly increased the proportion of GAD65-GFP boutons that colocalized with a newly formed Halo-Gephyrin scaffold (*p < 0.05, Fisher's exact test). ***iii***, There was no significant difference in the frequency of colocalization events with an existing Halo-Gephyrin scaffold in Sema4D versus control-treated cultures (*p* = 0.2048). ***C***, Mean velocity of GAD65-GFP boutons that colocalized with a newly formed Halo-Gephyrin scaffold does not differ from mean velocity of other GAD65-GFP boutons in control neurons (*p* = 0.960, unpaired heteroscedastic *t* test) or in Sema4D-treated neurons (*p* = 0.510). *n* = number of puncta per condition; error bars indicate SEM. ***D***, Initial distance to nearest gephyrin neighbor is smaller for GAD65-GFP boutons that colocalized with a newly formed Halo-Gephyrin scaffold compared with other GAD65-GFP boutons in both control neurons (***p < 0.001, unpaired heteroscedastic *t* test) and in Sema4D-treated neurons (***p < 0.001). There was no effect of Sema4D treatment for GAD65-GFP boutons that colocalized with a newly formed gephyrin scaffold in control versus Sema4D-treated cultures (*p* = 0.469). *n* = number of puncta per condition; error bars indicate SEM. ***E***, Mean GAD65-GFP bouton velocity was significantly decreased for GAD65-GFP boutons that colocalized with an existing Halo-Gephyrin scaffold compared with GAD65-GFP boutons without a new colocalization event (**p < 0.01). *n* = number of puncta per condition; error bars indicate SEM. ***F***, Initial distance to nearest gephyrin neighbor is smaller for GAD65-GFP boutons that colocalized with an existing Halo-Gephyrin scaffold compared with GAD65-GFP boutons without a new colocalization event (***p < 0.001, unpaired heteroscedastic *t* test). *n* = number of puncta per condition; error bars indicate SEM.

New colocalization events fell into two distinct categories: colocalization events between existing GAD65-GFP boutons and stable, preexisting gephyrin scaffolds (“existing gephyrin”; Fig. 3*A*) and colocalization events between existing GAD65-GFP boutons and newly formed Halo-Gephyrin scaffolds (“new gephyrin”; Fig. 3*B*). We found that in both control and Sema4D-treated neurons, colocalization events with new gephyrin scaffolds comprised ∼75% of the total new colocalization events (Fig. 4*A*). Sema4D treatment significantly increased the frequency of colocalization events between GAD65-GFP and new gephyrin scaffolds but not between GAD65-GFP and existing gephyrin (Fig. 4*Bii*–*iii*). These data suggest that one way in which colocalized gephyrin fluorescence is increased in response to Sema4D treatment is via new colocalization events between new gephyrin scaffolds and existing GAD65-GFP boutons.

### GAD65-GFP boutons that undergo new colocalization events occur in gephyrin-rich regions

Only a small subset of GAD65-GFP boutons underwent new colocalization events (Fig. 4*B*). What determines whether a specific GAD65-GFP bouton is “eligible” to participate in the assembly of a new synapse? We asked whether these boutons differ meaningfully from other GAD65-GFP boutons in specific properties such as mean velocity or initial proximity to gephyrin puncta at the outset of imaging. We first looked at GAD65-GFP boutons that colocalized with newly formed gephyrin scaffolds, as these comprised the majority of new colocalization events in both treatment conditions (Fig. 4*A*). Mean velocity did not differ between GAD65-GFP boutons that underwent colocalization events and GAD65-GFP boutons that did not, nor was there a Sema4D-dependent effect on mean velocity (Fig. 4*C*). In each treatment condition, mean initial nearest neighbor distance to gephyrin was significantly lower for GAD65-GFP boutons with a new colocalization event, suggesting that even though these boutons colocalized with gephyrin scaffolds that emerged during the imaging session, local proximity to other existing gephyrin scaffolds at the onset of imaging was strongly predictive of new colocalization events (Fig. 4*D*). This suggests that GAD65 puncta undergoing colocalization events are localized to “hot spot” regions of synapse assembly where more gephyrin is present to be recruited from nearby sites to form new synapses.

We performed the same analysis for new colocalization events in which GAD65-GFP boutons colocalized with an existing Halo-Gephyrin scaffold. These events were relatively less common, only comprising ∼25% of new colocalization events (Fig. 4*A*); due to the smaller sample size, we could not separately analyze control and Sema4D treatment conditions for this subset of events. These GAD65-GFP boutons had significantly lower mean velocity compared with boutons without a colocalization event (Fig. 4*E*), and initial nearest neighbor distance to existing gephyrin scaffolds was zero in every case in which we observed a new colocalization event (Fig. 4*F*), suggesting that these GAD65 boutons were initially colocalized with gephyrin, temporarily lost contact, and then colocalized again.

Taken together, these results suggest that Sema4D treatment increases the overall probability of new colocalization events, primarily by enhancing the formation of stable association between GAD65-GFP boutons and newly formed gephyrin scaffolds. Putative new synapse formation events involving GAD65-GFP boutons largely occur in gephyrin-rich regions where eligible postsynaptic sites are already nearby, even in cases where assembly of entirely new postsynaptic specializations precedes colocalization. In alignment with our previous findings that synaptic protein clusters rarely moved more than a few microns, these results support a model in which rapid inhibitory synapse assembly relies on local recruitment of protein clusters that are already poised for synaptogenesis.

### Sema4D treatment mobilizes GAD65-GFP boutons prior to colocalization with Halo-Gephyrin

**Figure 5.**
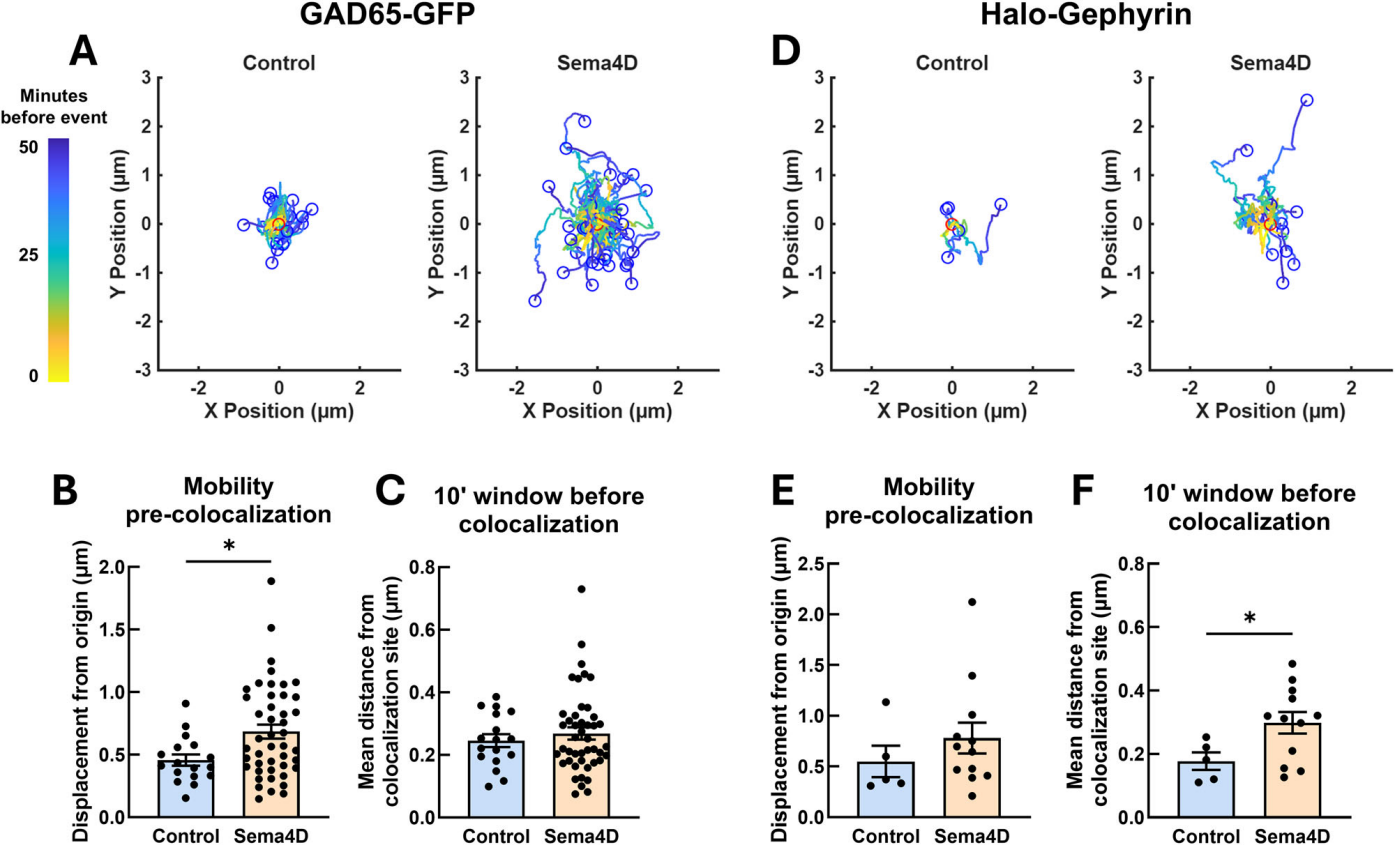
GAD65-GFP boutons mobilize before proximity-dependent recruitment of gephyrin scaffolds. ***A***, Tracks followed by GAD65-GFP boutons prior to new colocalization events with gephyrin in control (left) or Sema4D (right)-treated cultures. Blue dots represent relative start locations; red dots (at 0,0) represent normalized location of new colocalization event. ***B***, Total displacement of GAD65-GFP boutons undergoing a new colocalization event from *t* = 0 min until colocalization (*p < 0.05, Mann–Whitney *U* test). *n* = 17 events (control), 45 events (Sema4D). Error bars indicate SEM. ***C***, Mean distance of GAD65-GFP boutons from new colocalization sites in the 10 min window preceding new colocalization events. Sema4D treatment did not affect mean GAD65-GFP distance during this time window (*p* = 0.8513, Mann–Whitney *U* test). *n* = 17 events (control), 45 events (Sema4D). Error bars indicate SEM. ***D***, Tracks followed by Halo-Gephyrin scaffolds from *t* = 0 min until colocalization with GAD65-GFP in control (left) or Sema4D (right)-treated cultures. Blue dots represent relative start locations; red dots (0,0) represent normalized location of new colocalization event. ***E***, Total displacement of Halo-Gephyrin scaffolds undergoing a new colocalization event from *t* = 0 min until colocalization. Sema4D treatment does not affect displacement of Halo-Gephyrin scaffolds undergoing colocalization events (*p* = 0.2343, Mann–Whitney *U* test). *n* = 5 events (control), 12 events (Sema4D). Error bars indicate SEM. ***F***, In Sema4D-treated cultures, Halo-Gephyrin scaffolds are on average significantly farther from new colocalization sites during the 10 min window preceding new colocalization events compared with control cultures (*p < 0.05, Mann–Whitney *U* test). *n* = 5 events (control), 12 events (Sema4D). Error bars indicate SEM.

Given that Sema4D increases the overall mobility of GAD65-GFP but not Halo-Gephyrin puncta (Fig. 2) and that Sema4D increases the probability of new colocalization events between GAD65 and gephyrin (Figs. 3, 4), we hypothesized that presynaptic protein clusters define the sites of new GABAergic synapses. To test this directly, we performed an event-level analysis of paths followed by GAD65-GFP or Halo-Gephyrin puncta before and after new colocalization events (Fig. 5*A*,*D*). For this analysis, all new colocalization events (including those involving new and existing gephyrin scaffolds) were combined for each treatment condition. We analyzed two key time windows: first, the entire window from the beginning of the imaging session until the new colocalization event occurred, and second, the specific 10 min window just prior to the colocalization event. We reasoned that if one synaptic partner consistently established the sites at which new colocalization events were to occur, it would show greater mobility in the earlier window than the later window, indicating it had moved and then stopped at a specific location. On the other hand, if one synaptic partner was recruited only once in proximity to an established site, it would be farther from colocalization sites during the later 10 min window, indicating it was rapidly recruited to the nascent synapse just prior to colocalizing. GAD65-GFP boutons that colocalized with Halo-Gephyrin scaffolds displaced significantly farther from their origin prior to colocalization in Sema4D-treated cultures compared with control (Fig. 5*B*). When we examined the mean distance of GAD65-GFP boutons from the site of colocalization specifically in the 10 min window just prior to colocalization events there was no difference between control and Sema4D-treated cultures (Fig. 5*C*). This suggests that Sema4D-dependent changes to presynaptic mobility occur in an earlier window relative to when GAD65-GFP boutons become colocalized with gephyrin.

We next analyzed the characteristics of Halo-Gephyrin scaffolds that became colocalized with a GAD65-GFP bouton, focusing specifically on this subset to examine postsynaptic protein cluster dynamics at sparsely labeled presynaptic sites. In contrast to our observations for GAD65-GFP boutons, Sema4D treatment had no effect on overall Halo-Gephyrin scaffold displacement across the entire time window before colocalization (Fig. 5*E*), consistent with the lack of Sema4D-dependent effect on postsynaptic mobility overall (Fig. 2). However, in contrast to GAD65-GFP boutons, Halo-Gephyrin scaffolds were on average farther from the colocalization site in the 10 min window prior to colocalization in Sema4D-treated cultures compared with control (Fig. 5*F*). This suggests that gephyrin scaffold mobility is influenced by Sema4D signaling in the immediate window prior to colocalization, but not before, possibly because the presence of a nearby presynaptic bouton is required for the gephyrin scaffold to mobilize. Taken together, these results suggest that GAD65-GFP mobility occurs first in the sequence of events underlying Sema4D-dependent synapse formation, followed by later gephyrin recruitment, and that mobile GAD65-GFP boutons likely encounter gephyrin assemblies stochastically.

### Sema4D drives GABA_A_R recruitment to preexisting but immature postsynaptic gephyrin scaffolds

GABAergic synapse maturation requires the precise recruitment and stabilization of GABA_A_Rs at postsynaptic gephyrin scaffolds, and gephyrin has been shown to regulate both GABA_A_R clustering and surface expression (Essrich et al., 1998; Mukherjee et al., 2011; Petrini et al., 2014). Whether this scaffold–receptor relationship is established in concert with other synaptogenic processes or whether receptor clustering and scaffold assembly are independent events remains an open question. Previous work from our lab demonstrated that Sema4D increases the density of GABA_A_Rγ2-positive synapses and that newly formed synapses are functional within 2 h (Kuzirian et al., 2013), raising the possibility that functional GABA_A_Rs are localized to nascent synaptic sites almost immediately upon their formation. Yet whether gephyrin and GABA_A_Rs are recruited simultaneously or consecutively in this context has not been directly examined, leaving the spatiotemporal logic of Sema4D-induced synapse assembly unresolved.

**Figure 6.**
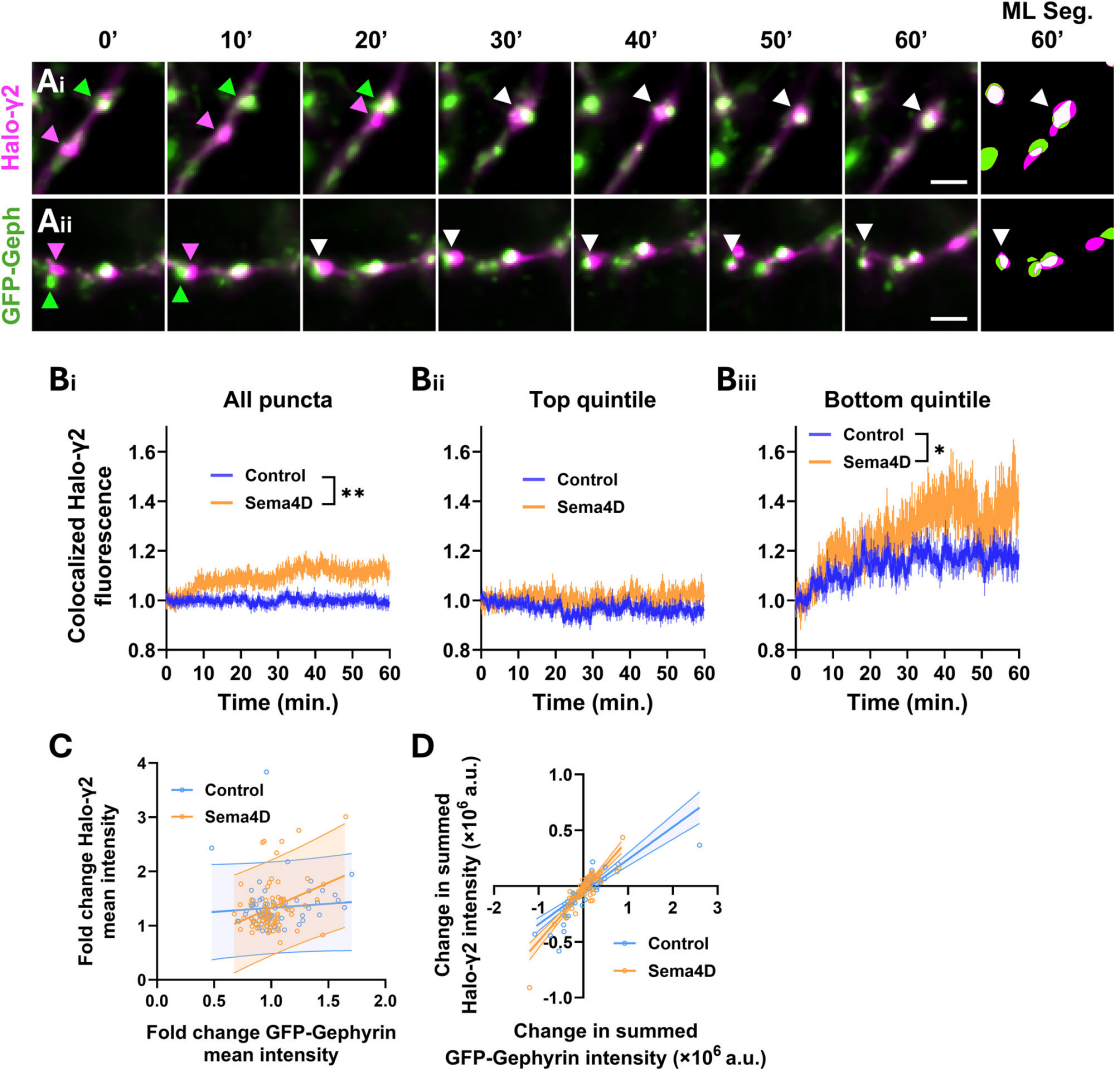
Sema4D promotes recruitment of GABA_A_Rs to postsynaptic gephyrin scaffolds and increases the receptor-binding capacity of scaffolds. ***A***, Representative images showing colocalization between GFP-Gephyrin (green arrows) and Halo-γ2 (magenta arrows). ***i***, ***ii*** Examples showing a Halo-γ2 puncta moving and colocalizing with a GFP-Gephyrin scaffold (white arrows, colocalization). Images represent different regions from the same neuron. Scale bars, 2 µm. ***B***, ***i***, Sema4D treatment increases mean Halo-γ2 intensity colocalized with GFP-Gephyrin scaffolds compared with control treatment (binned LME, time × treatment interaction, *F*_(1,4964)_ = 8.5686; **p < 0.01). *n* ≥ 254 puncta per timepoint (control), 130 puncta (Sema4D). ***ii***, Sema4D treatment does not affect mean Halo-γ2 intensity colocalized with GFP-Gephyrin scaffolds in the top quintile of baseline Halo-γ2 signal compared with control treatment (binned LME: time × treatment interaction: *F*_(1,992)_ = 1.1088, *p* = 0.2926). *n* ≥ 48 puncta per timepoint (control), 24 puncta (Sema4D). ***iii***, Sema4D treatment increases mean Halo-γ2 intensity colocalized with GFP-Gephyrin scaffolds in the bottom quintile of baseline Halo-γ2 intensity compared with control treatment (binned LME, time × treatment interaction, *F*_(1,992)_ = 4.2854; *p < 0.05). *n* ≥ 49 puncta per timepoint (control), 24 puncta (Sema4D). Error bars indicate SEM. Data are normalized within treatment condition to the mean of the first 3 min. ***C***, Fold change in Halo-γ2 mean intensity as a function of fold change in GFP-Gephyrin mean intensity at colocalized puncta. Control neurons show no significant relationship (95% CI of slope, −0.2897 to 0.5903; *F*_(1,68)_ = 0.4646; *p* = 0.4978), whereas Sema4D-treated neurons show a significant positive correlation (95% CI of slope, 0.3273–1.520; *F*_(1,74)_ = 9.527; *p* < 0.01). *n* = 70 puncta (control), 76 puncta (Sema4D). ***D***, Change in summed Halo-γ2 intensity as a function of change in summed GFP-Gephyrin intensity at the same puncta. Both conditions show strong positive relationships (control 95% CI of slope, 0.2367–0.3441; *F*_(1,68)_ = 116.5; *p* < 0.0001; Sema4D 95% CI of slope, 0.3944–0.5138; *F*_(1,76)_ = 229.9; *p* < 0.0001), but the slope is steeper in Sema4D-treated neurons (ANCOVA, *F*_(1,142)_ = 15.38; *p* < 0.0001), indicating that Halo-γ2 accumulation outpaces gephyrin accumulation under Sema4D. *n* = 70 puncta (control), 76 puncta (Sema4D). Refer to Extended Data Figure 6-1 for additional analysis of GFP-Gephyrin/Halo-γ2 colocalization. Refer to Extended Data Figures 6-2 and 6-3 for quantification of GABAergic synapse density in cells coexpressing GFP-Gephyrin and Halo-γ2.

To begin to address this question, we cotransfected plasmids expressing GFP-Gephyrin and HaloTag-GABA_A_Rγ2 subunit (Halo-γ2) into cultured wild-type E18 rat neurons at DIV4 and performed live imaging at DIV10–11 as before using JF646 HaloTag ligand to label the Halo-γ2 protein (Fig. 6*A*; Extended Data Fig. 6-1). As with the virally expressed Halo-Gephyrin construct, we observed that GFP-Gephyrin formed discrete clusters, whereas Halo-γ2 expression showed both punctate and diffuse expression consistent with incorporation into synaptic and extrasynaptic receptor pools (Extended Data Fig. 6-1). GFP-Gephyrin and Halo-γ2 puncta generally colocalized, and fixed immunostaining experiments showed that overexpression of these markers did not affect baseline GABAergic synapse density or interfere with synaptic localization of Halo-γ2 puncta (Extended Data Figs. 6-2, 6-3).

10.1523/ENEURO.0140-26.2026.f6-1 Figure 6-1 **Sema4D does not affect mean Halo-γ2 puncta intensity or total dendritic intensity or GFP-Gephyrin size or intensity; independent Halo-γ2 puncta are more mobile than scaffold-associated puncta.** (A) Representative regions showing expression pattern of GFP-Gephyrin (green) and Halo-γ2 (magenta) along dendrites of cultured E18 rat neurons. Most Halo-γ2 clusters are colocalized with GFP-Gephyrin (white arrows), but some independent clusters are observed (magenta arrows). Note diffuse extrasynaptic expression of Halo-γ2 along dendrite. Images represent different regions from the same neuron. Scale bar = 5 µm. (B) There was no effect of Sema4D treatment on mean intensity of Halo-γ2 puncta (binned LME: time × treatment interaction: F(1, 1508) = 0.0017 , p = 0.9670). n ≥ 69 puncta per timepoint (control), 45 puncta (Sema4D). Error bars = SEM. Data are normalized within treatment condition to the mean of the first 3 minutes. (C) There was no effect of Sema4D treatment on mean total dendritic expression of Halo-γ2 (binned LME: time × treatment interaction: F(1, 140) = 1.3144 , p = 0.2536). n = 7 cells (control), 5 cells (Sema4D). Error bars = SEM. (D) Sema4D does not affect mean area of GFP-Gephyrin that recruit Halo-γ2 compared to control (Mann-Whitney U-test, p = 0.8720). n = 70 puncta (control), 76 puncta (Sema4D). (E) Sema4D does not affect mean summed intensity of GFP-Gephyrin that recruit Halo-γ2 compared to control (Mann-Whitney U-test, p = 0.7208). n = 70 puncta (control), 76 puncta (Sema4D). (F) Mean velocity is significantly greater for Halo-γ2 puncta that are not colocalized with GFP-Gephyrin in both control (***p < 0.001, unpaired heteroscedastic t-test) and Sema4D-treated neurons (***p < 0.001). Download Figure 6-1, TIF file.

10.1523/ENEURO.0140-26.2026.f6-2 Figure 6-2 **Overexpression of GFP-Gephyrin increases gephyrin puncta density but does not affect GABAergic synapse density; Halo-γ2 overexpression does not affect gephyrin puncta or synapse density.** (A) Sample stretches of dendrite from neurons co-expressing TdTomato (red) with indicated constructs. Scale bar = 5 µm. (B) GFP-Gephyrin overexpression (OE) increases gephyrin puncta density on cultured neurons compared to empty vector (EV) control transfection (p = 0.0027, Tukey post-hoc), whereas Halo-γ2 OE does not affect gephyrin puncta density (p > 0.99). Co-expression of GFP-Gephyrin and Halo-γ2 does not increase gephyrin puncta density more than GFP-Gephyrin alone (p = 0.95). (C) Overexpression of GFP-Gephyrin alone, Halo-γ2 alone, or combined overexpression of GFP-Gephyrin + Halo-γ2 does not affect density of GAD65 puncta (F(3, 59) = 2.256, p = 0.0912). (D) Overexpression of GFP-Gephyrin alone, Halo-γ2 alone, or combined overexpression does not affect GAD65/gephyrin synapse density compared to EV control transfection (F(3, 59) = 1.676, p = 0.536, ordinary one-way ANOVA). Download Figure 6-2, TIF file.

10.1523/ENEURO.0140-26.2026.f6-3 Figure 6-3 **GFP-Gephyrin overexpression does not affect synaptic localization of Halo-γ2.** (A) Co-expression of GFP-Gephyrin + Halo-γ2 increases the density of Halo-γ2 compared to Halo-γ2 OE alone (p = 0.0143, unpaired t-test). (B) GFP-Gephyrin OE does not affect density of GAD65+ inputs to transfected cells (p = 0.6309). (C) Co-expression of GFP-Gephyrin and Halo-γ2 does not affect Halo-γ2+ synapse density compared to Halo-γ2 OE alone (p = 0.436). Download Figure 6-3, TIF file.

We first analyzed the mean fluorescence intensity of Halo-γ2 colocalized with GFP-Gephyrin as a measure of receptor density at postsynaptic scaffolds (Fig. 6*B*), similar to our previous analysis of GAD65 and gephyrin colocalization (Fig. 3). We found that the mean colocalized Halo-γ2 fluorescence rapidly increased in response to Sema4D treatment within ∼10 min of application. By 1 h, the mean increase in colocalized Halo-γ2 fluorescence at GFP-Gephyrin puncta was ∼10–15% above baseline in Sema4D-treated cultures (Fig. 6*Bi*). Sema4D treatment did not affect the mean intensity of individual Halo-γ2 puncta or the total integrated intensity of Halo-γ2 in dendrites (Extended Data Fig. 6-1*B*,*C*); thus, the Sema4D-dependent increase in colocalized Halo-γ2 fluorescence is likely due to increased recruitment of Halo-γ2 to gephyrin-positive postsynaptic scaffolds rather than increased GABA_A_R expression.

Is the observed Sema4D-driven Halo-γ2 recruitment simply a downstream consequence of expanded gephyrin scaffolds? We addressed this possibility in two ways: first, we examined whether Sema4D affects the size or summed fluorescence intensity of GFP-Gephyrin scaffolds that recruited GABA_A_Rs. There was no significant effect of Sema4D on either parameter (Extended Data Fig. 6-1*D*,*E*), indicating that gephyrin scaffolds that recruited receptors were structurally unchanged. Second, we examined the relationship between gephyrin and GABA_A_R changes at individual postsynaptic sites where GABA_A_Rs were recruited. In control neurons, the fold change in mean Halo-γ2 intensity was uncorrelated with fold change in mean GFP-Gephyrin intensity (Fig. 6*C*), while summed Halo-γ2 and GFP-Gephyrin intensities scaled in tight proportion (Fig. 6*D*). This pattern is consistent with the established near-1:1 stoichiometry of GABA_A_Rs to gephyrin molecules at mature inhibitory synapses (Fritschy et al., 2012; Specht et al., 2013): as gephyrin scaffolds grow, receptors are recruited proportionally, leaving local receptor density unchanged. Sema4D treatment disrupted this proportional relationship: changes in mean Halo-γ2 intensity scaled positively with changes in mean GFP-Gephyrin intensity (Fig. 6*C*), and summed Halo-γ2 intensity accumulated at a superlinear rate relative to summed GFP-Gephyrin intensity (Fig. 6*D*). Thus, Sema4D does not drive GABA_A_R recruitment merely by enlarging gephyrin scaffolds; rather, Sema4D increases the number of GABA_A_ receptors recruited per unit of gephyrin scaffold without altering overall scaffold structure.

How does Sema4D drive GABA_A_R recruitment to the postsynaptic scaffold? Two broad mechanisms are possible: capture of diffuse or mobile GABA_A_Rs by established gephyrin scaffolds or coalescence of preassembled gephyrin and GABA_A_R clusters that encounter one another. If the first mechanism operates, a further question arises—whether recruitment efficiency depends on the amount of gephyrin already present at a given scaffold. Thus, we next asked whether Sema4D preferentially recruits GABA_A_Rs to gephyrin scaffolds that are receptor-poor at baseline. We analyzed the mean colocalized Halo-γ2 fluorescence in the subset of GFP-Gephyrin scaffolds in the top quintile of baseline Halo-γ2 fluorescence versus GFP-Gephyrin scaffolds in the bottom quintile of baseline Halo-γ2 fluorescence. Sema4D treatment significantly increased colocalized Halo-γ2 fluorescence at gephyrin scaffolds in the lowest quintile of baseline receptor expression (Fig. 6*Biii*) while having no effect on gephyrin scaffolds in the top quintile (Fig. 6*Bii*). Together these results suggest that Sema4D increases Halo-γ2 localization at gephyrin-labeled scaffolds primarily by redistributing existing receptors to scaffolds lacking GABA_A_Rs rather than by altering overall Halo-γ2 expression levels or receptor density at existing postsynaptic sites.

While most clustered GABA_A_Rs were localized to postsynaptic specializations marked by GFP-Gephyrin, a subset of these clusters appeared to move independently of gephyrin. These Halo-γ2 puncta were significantly more mobile than those associated with GFP-Gephyrin, suggesting that they are less constrained and therefore unlikely to be colocalized with unlabeled endogenous scaffolds (Extended Data Fig. 6-1*F*). Notably, preclustered GABA_A_R assemblies that are not associated with gephyrin have been described previously in fixed immunostaining experiments with endogenous proteins (Christie et al., 2002; 2006; Danglot et al., 2003), but whether and how they eventually localize to synapses is unclear. We therefore asked whether the independent Halo-γ2 puncta we observed represented a pool of preassembled receptor clusters poised to coalesce with gephyrin scaffolds and whether Sema4D drives this process.

**Figure 7.**
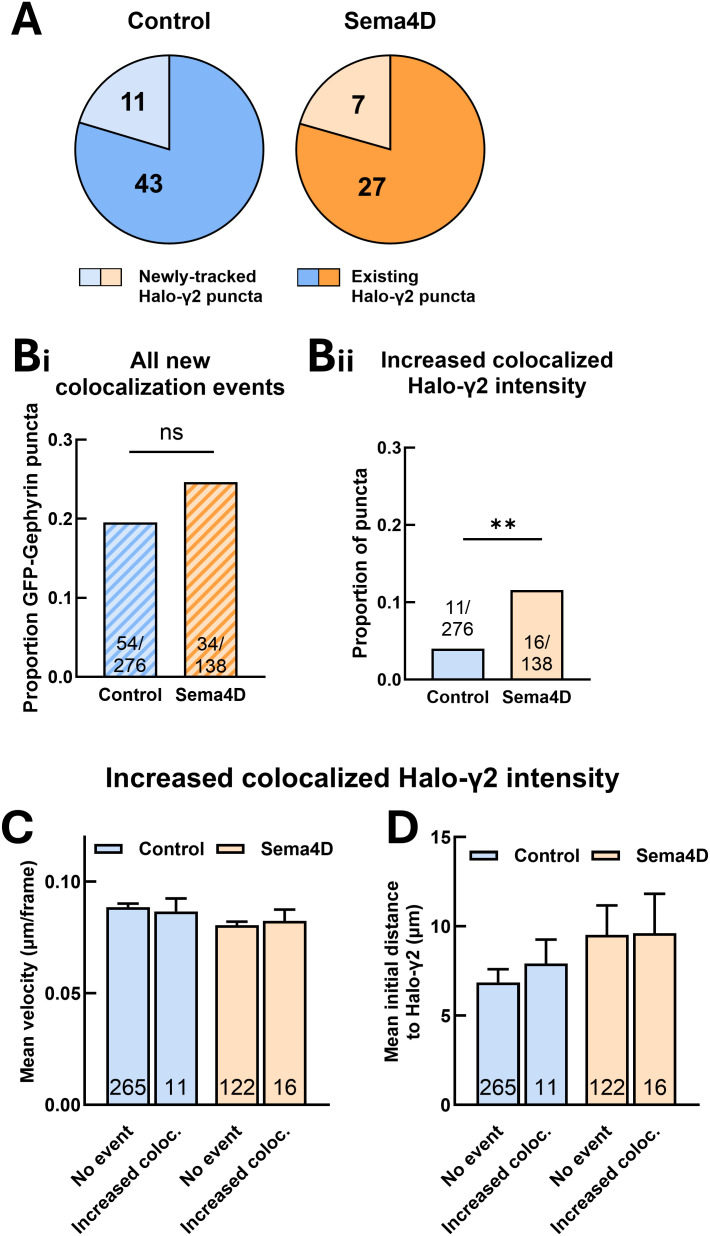
Sema4D-dependent GABA_A_R recruitment occurs through gradual receptor accumulation rather than capture of preclustered receptor assemblies. ***A***, Number of new colocalization events between GFP-Gephyrin and existing Halo-γ2 puncta versus newly formed Halo-γ2 puncta. ***B***, ***i***, Overall proportion of GFP-Gephyrin scaffolds with a new colocalization event. There was no effect of Sema4D treatment on the proportion of GFP-Gephyrin scaffolds with any type of new colocalization event (*p* = 0.2525). ***ii***, Proportion of GFP-Gephyrin scaffolds with ≥1.5-fold increase in colocalized Halo-γ2 fluorescence. Sema4D treatment significantly increased the proportion of GFP-Gephyrin scaffolds with increased Halo-γ2 fluorescence (**p < 0.01, Fisher's exact test). ***C***, Mean velocity of GFP-Gephyrin scaffolds with a ≥1.5-fold increase in Halo-γ2 fluorescence does not differ from mean velocity of other GFP-Gephyrin scaffolds in control neurons (*p* = 0.760, unpaired heteroscedastic *t* test) or in Sema4D-treated neurons (*p* = 0.727). *n* = number of puncta per condition; error bars indicate SEM. ***D***, Initial nearest neighbor distance to Halo-γ2 puncta is not significantly different for GFP-Gephyrin scaffolds with increased Halo-γ2 fluorescence compared with other GFP-Gephyrin scaffolds in control neurons (*p* = 0.503, unpaired heteroscedastic *t* test) or in Sema4D-treated neurons (*p* = 0.144). *n* = number of puncta per condition; error bars indicate SEM.

Similar to our previous analysis of GAD65 and gephyrin colocalization (Fig. 4), we identified GFP-Gephyrin scaffolds that did not initially cocluster with GABA_A_R but became colocalized during the imaging session and remained stably colocalized for at least 10 min (representing new colocalization events). We found that in contrast to GAD65 and gephyrin, in which the majority of new colocalization events involved emergence of a new gephyrin scaffold, most new colocalization events between GFP-Gephyrin and Halo-γ2 puncta (∼80% of events) were between pairs of preexisting puncta (Fig. 7*A*). Surprisingly, however, new colocalization events occurred at a similar frequency in both control and Sema4D-treated neurons (Fig. 7*Bi*), suggesting that Sema4D does not drive localization of independent GABA_A_R clusters to postsynaptic scaffolds. Thus, the occurrence of new colocalization events between GFP-Gephyrin and Halo-γ2 puncta fails to explain the Sema4D-dependent increase in total Halo-γ2 recruitment.

### Recruitment of GABA_A_Rs that were not previously associated with GFP-Gephyrin scaffolds underlies Sema4D-dependent postsynaptic maturation

Since Sema4D treatment did not drive GFP-Gephyrin scaffolds to recruit preformed Halo-γ2 clusters, we identified a separate population of GFP-Gephyrin scaffolds in which colocalized Halo-γ2 fluorescence increased at least 1.5-fold during the imaging session regardless of whether a new colocalization event with a preassembled receptor cluster occurred. This separate population of postsynaptic scaffolds presumably represents sites in which Sema4D drives accumulation of receptors from diffuse or mobile receptor pools. We found that, compared with control, Sema4D significantly increased the fraction of gephyrin scaffolds that underwent at least a 1.5-fold increase in colocalized Halo-γ2 fluorescence, from ∼4 to 11.5% (Fig. 7*Bii*). Next, to understand which postsynaptic sites are targeted for Sema4D-dependent receptor recruitment, we asked whether these GFP-Gephyrin scaffolds differed intrinsically from scaffolds that did not recruit GABA_A_Rs. Surprisingly, GFP-Gephyrin scaffolds that recruited receptors did not differ from other GFP-Gephyrin scaffolds in their mean velocity (Fig. 7*C*) or proximity to Halo-γ2 puncta (Fig. 7*D*), nor did they differ between treatment conditions. This suggests sites of Sema4D-dependent GABA_A_R recruitment are not necessarily in regions of the dendrite where preclustered receptor assemblies are already present, instead drawing on diffuse receptor pools. Together, these data indicate that coalescence of independent GABA_A_R clusters with gephyrin scaffolds is a constitutive feature of postsynaptic assembly rather than a Sema4D-regulated process. Combined with the preferential recruitment of receptors to receptor-poor scaffolds (Fig. 6*Biii*), these findings support a model in which Sema4D drives GABA_A_R recruitment primarily through capture of diffuse or mobile receptors by established gephyrin scaffolds rather than by directing the coalescence of preassembled receptor clusters.

### New inhibitory postsynaptic sites arise from preexisting clusters of either gephyrin scaffold or GABA_A_Rs

**Figure 8.**
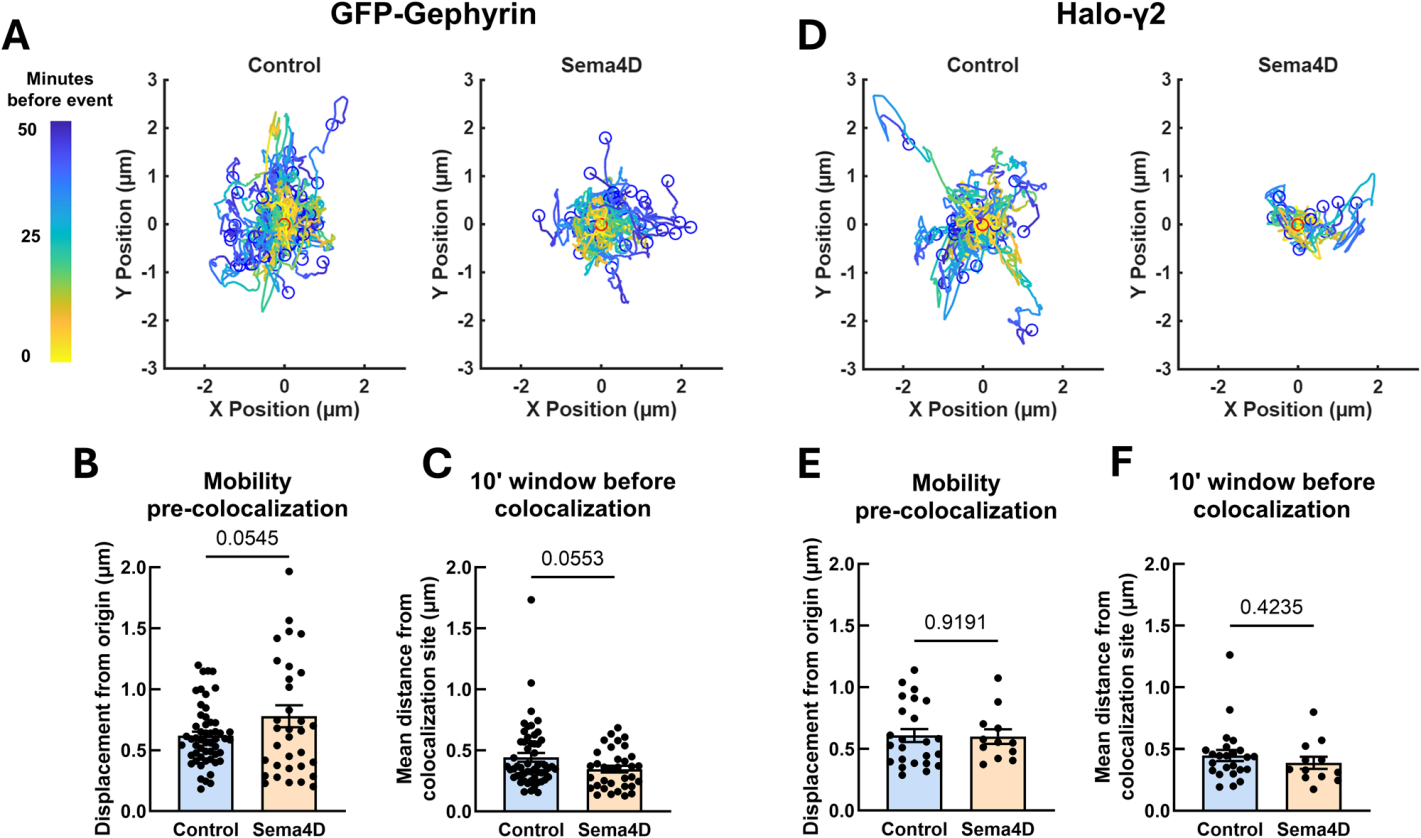
Both GFP-Gephyrin scaffolds and preclustered GABA_A_R assemblies can establish new postsynaptic sites. ***A***, Tracks followed by GFP-Gephyrin scaffolds prior to new colocalization events with Halo-γ2 puncta in control (left) or Sema4D (right)-treated cultures. Blue dots represent relative start locations; red dots (0,0) represent normalized location of new colocalization event. ***B***, Displacement distance of GFP-Gephyrin scaffolds undergoing a new colocalization event prior to colocalizing. Comparison shows displacement from origin of GFP-Gephyrin scaffolds that colocalized with a Halo-γ2 puncta in Sema4D-treated cultures compared with control (*p* = 0.0545, Mann–Whitney *U* test). ***C***, Mean distance of GFP-Gephyrin scaffolds from new colocalization sites in the 10 min window preceding new colocalization events. Comparison shows mean distance of GFP-Gephyrin scaffolds undergoing colocalization events with Halo-γ2 puncta in Sema4D-treated cultures compared with control (*p* = 0.0553). *n* = 54 events (control), 34 events (Sema4D). Error bars represent SEM. ***D***, Tracks followed by Halo-γ2 puncta prior to new colocalization events in control (left) or Sema4D (right)-treated cultures. Blue dots represent relative start locations; red dots (0,0) represent normalized location of new colocalization event. ***E***, Displacement distance of Halo-γ2 puncta undergoing a new colocalization event with GFP-Gephyrin prior to colocalization. Sema4D treatment does not affect displacement of Halo-γ2 puncta undergoing colocalization events with GFP-Gephyrin scaffolds (*p* = 0.9191, Mann–Whitney *U* test). ***F***, Sema4D treatment does not affect mean distance of Halo-γ2 puncta from colocalization sites during the 10 min window preceding new colocalization events compared with control (*p* = 0.4235). *n* = 24 events (control), 12 events (Sema4D). Error bars represent SEM.

A long-standing model of GABAergic synapse maturation focuses on the behavior of individual receptors which diffuse in the membrane and can be captured and confined at gephyrin scaffolds, thus localizing them to postsynaptic specializations. Our live-imaging approach offered an opportunity to gain insight into this process by tracking the behavior of both GFP-Gephyrin and Halo-γ2 puncta in the moments leading up to new colocalization events. We analyzed the paths followed by GFP-Gephyrin and Halo-γ2 puncta prior to new colocalization events, beginning by tracking the total displacement distance of GFP-Gephyrin and Halo-γ2 clusters in the entire time window leading up to new colocalization events (Fig. 8*A*,*D*). In control-treated neurons, we observed that, surprisingly, GFP-Gephyrin and Halo-γ2 puncta moved similar distances from their original location prior to colocalization, with few puncta moving >2 µm prior to colocalization in all conditions (Fig. 8*B*,*E*). These data suggest that either gephyrin or GABA_A_R cluster mobility can promote a new colocalization event. We observed that Sema4D treatment led to a marginally significant increase in the mean distance traveled by GFP-Gephyrin scaffold assemblies prior to colocalizing (*p* = 0.0545; Fig. 8*B*) and a marginally significant decrease in the average distance of GFP-Gephyrin to colocalization sites in the 10 min window prior to colocalization (*p* = 0.0553; Fig. 8*C*). This suggests that Sema4D treatment increases mobility of this subset of GFP-Gephyrin scaffolds earlier than 10 min before colocalization with preclustered Halo-γ2 puncta.

We performed similar analyses on Halo-γ2 mobility before and after colocalization with GFP-Gephyrin and found no difference in any of these parameters between control and Sema4D-treated cultures (Fig. 8*E*,*F*). Taken together these data suggest that, contrary to the canonical model, both gephyrin scaffolds and preclustered GABA_A_R assemblies are mobile and are equally capable of initiating a colocalization event. Notably, because this analysis is restricted to receptor clusters large enough to be resolved as discrete puncta by confocal microscopy, it does not capture the behavior of diffusely distributed or subpuncta receptor populations, which may follow different dynamics. Nevertheless, these new colocalization events occur at a substantial proportion of gephyrin scaffolds and appear to be stochastic events that do not require Halo-γ2 puncta to be recruited to specific preestablished sites. Overall, these experiments suggest a model in which mobile preformed clusters of gephyrin and GABA_A_Rs may encounter each other to form new postsynaptic specializations. While Sema4D modulates gephyrin mobility prior to colocalization, the overall rate of colocalization events is not significantly enhanced; rather, enhanced recruitment or capture of individual GABA_A_Rs by relatively immature postsynaptic scaffolds is the primary mechanism which drives Sema4D-mediated receptor recruitment and synapse maturation.

## Discussion

Compared with excitatory synapse formation, inhibitory synapse formation has historically been more challenging to study due to the lack of an extensive postsynaptic density for biochemical purification and the relatively low abundance of inhibitory synapses. The spatiotemporal dynamics of inhibitory synapse assembly are poorly understood, particularly at acute timescales, as the vast majority of assays employed to study the function of synaptogenic molecules have assessed synapse formation by performing a manipulation (e.g., gene knock-out) and assaying the presence or absence of synapses by microscopy or electrophysiology. These retrospective approaches likely obscure significant nuances in the spatiotemporal dynamics of synaptic protein cluster formation, stabilization at sites of colocalization, and maturation. Thus there remains a significant gap in understanding the process by which molecular signals and cellular processes transform nascent contacts into mature inhibitory synapses.

Over the past decade, we defined the novel role of Class 4 Semaphorins and Plexin-B receptors in regulating inhibitory synapse formation in the rodent hippocampus. Specifically, our lab demonstrated that the soluble, extracellular domain of Sema4D induces inhibitory synapse formation on a rapid timescale (i.e., minutes) while having no effect on excitatory synapse formation. Among the handful of identified trans-synaptic regulators of GABAergic synapse assembly, which include the Neuregulin-ErbB4, Neuroligin-Neurexin, Slitrk-PTPδ, FGF, and Dystroglycan families (Levinson et al., 2005; Krivosheya et al., 2008; Takahashi et al., 2012; Yim et al., 2013; Dabrowski et al., 2015; Trotter et al., 2023), only Sema4D has these unique properties. This rapid, selective, and inducible effect enables precise dissection of synaptogenic mechanisms that are otherwise difficult to study due to the asynchronous and developmentally protracted nature of synaptogenesis.

**Figure 9.**
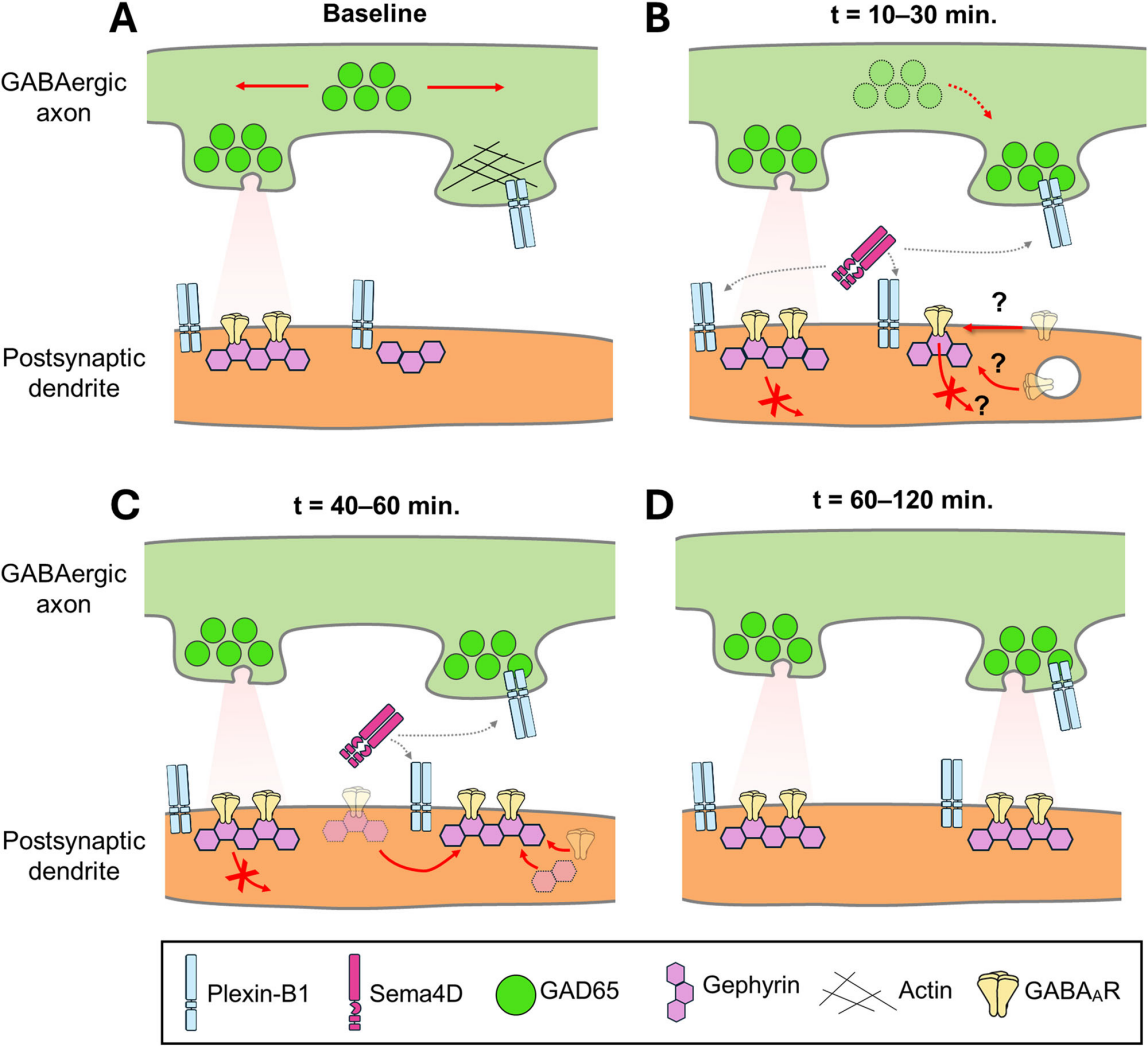
Proposed model of Sema4D-induced GABAergic synapse formation. ***A***, Presynaptic neuron and postsynaptic neuron prior to Sema4D addition. Top, Presynaptic GAD65-containing boutons are mobile but are constrained by actin assemblies. Bottom, Postsynaptic dendrites contain mature postsynaptic scaffolds (left) and immature scaffolds lacking receptors (right). Red cone represents a site of GABA release. ***B***, Beginning around *t* = 10–30 min. Top, In the presynaptic neuron Sema4D signaling to Plexin-B1 promotes cytoskeletal disassembly, priming eventual presynaptic sites. This allows mobile GAD65-containing boutons to diffuse to a new axonal site, establishing a new presynaptic terminal. Bottom, In the postsynaptic neuron, Sema4D signals to Plexin-B1 to stabilize mature postsynaptic specializations (left) preventing disassembly. GABA_A_Rs begin to accumulate at immature gephyrin scaffolds initially lacking receptors (right), although the specific mechanisms by which these receptors are recruited are unknown. ***C***, By *t* = 40–60 min, colocalization between GAD65 and gephyrin increases, driven by accumulation of postsynaptic scaffolds near presynaptic boutons previously lacking gephyrin. Juxtacrine or paracrine Sema4D/Plexin-B1 signaling mobilizes gephyrin scaffolds specifically in the vicinity of a bouton, establishing trans-synaptic alignment. Recruitment of gephyrin and GABA_A_Rs to maturing postsynaptic sites occurs in parallel. ***D***, Transient contacts continue to mature and accumulate synaptic proteins. By *t* = 120 min. newly formed synapses are functional.

In this study, we leveraged the ability of Sema4D to induce GABAergic synapse formation on the scale of minutes coupled with two-channel live imaging to characterize the dynamics of synaptic proteins during Sema4D-mediated synapse assembly. Our data support a temporally ordered model of synapse formation in which Sema4D engages pre- and postsynaptic compartments through distinct but coordinated mechanisms (Fig. 9). Sema4D binding to Plexin-B1 initiates two early events in parallel: in the presynaptic neuron, Sema4D increases mobility of presynaptic boutons within ∼20 min (Fig. 2), followed by increased bouton stability (Figs. 1, 2*A*, 9*A*,*B*). In this model, increased presynaptic mobility allows GAD65 boutons to explore a wider radius, increasing the likelihood that a bouton will localize to a synaptic site where it becomes stabilized. Simultaneously, Sema4D drives GABA_A_R recruitment to postsynaptic scaffolds within ∼10 min (Fig. 6), preceding measurable changes in GAD65–gephyrin colocalization. This is consistent with a cell-autonomous postsynaptic priming mechanism that does not require trans-synaptic contact (Fig. 9*A*,*B*).

Importantly, Sema4D does not globally alter gephyrin mobility (Fig. 2); rather, gephyrin scaffold assemblies are mobilized only once in the immediate vicinity of a presynaptic bouton (Fig. 5), suggesting that postsynaptic recruitment at this stage is locally triggered by proximity to an eligible presynaptic partner. By 30–40 min, this coordinated sequence produces increased GAD65–gephyrin colocalization (Fig. 3), driven by accumulation of postsynaptic protein at presynaptic boutons that previously lacked gephyrin and stabilization of postsynaptic scaffolds at existing synapses (Figs. 4, 9*C*). Because this effect is proximity dependent, it is likely that juxtacrine or paracrine Sema4D/Plexin-B1 signaling in the vicinity of the bouton establishes transient trans-synaptic contacts that protect these boutons from elimination, thus stabilizing them at sites of putative new synapses. These contacts continue to mature and accumulate synaptic proteins, such that by 120 min, newly formed synapses are functionally inhibitory (Fig. 9*D*; Kuzirian et al., 2013; Adel et al., 2023). Our findings agree with converging evidence from multiple groups that the presynaptic proteins accumulate first at nascent GABAergic synapses during early development (Wierenga et al., 2008; Dobie and Craig, 2011; Kuriu et al., 2012). Taken together, these data suggest that Sema4D acts primarily on preexisting pools of synaptic proteins, mobilizing presynaptic boutons, priming postsynaptic scaffolds for receptor capture, and stabilizing transient contacts to assemble inhibitory synapses at acute timescales.

How are GABA_A_Rs recruited to gephyrin scaffolds in response to Sema4D? The canonical model of inhibitory postsynaptic assembly which emerged from early single-particle tracking and FRAP experiments posits that gephyrin scaffolds are stable structures which capture laterally diffusing GABA_A_Rs upon synaptic entry (Jacob et al., 2005). Subsequent biochemical work supported this anchoring model, showing that gephyrin and collybistin directly bind GABA_A_Rs to stabilize receptor localization (Tretter et al., 2008; Hines et al., 2018; Lorenz-Guertin and Jacob, 2018). Our live-imaging data support this general framework but reveal a more dynamic view of postsynaptic assembly. We found that Sema4D promotes recruitment of GABA_A_Rs to less mature gephyrin scaffolds with an abundance of available binding sites rather than enhancing receptor clustering at sites where many receptors are already present. Notably, Sema4D did not alter the frequency of new colocalization events between preassembled gephyrin and GABA_A_Rγ2 puncta (Fig. 8), indicating that the effect of Sema4D is primarily to stabilize or enhance accumulation of individual receptors at postsynaptic sites lacking receptors.

Interestingly, we observed that both gephyrin and GABA_A_Rγ2 could establish new sites of colocalization by moving toward putative new postsynaptic sites and that this process plays an important role in postsynaptic assembly during development. Recent work suggests that gephyrin turnover and assembly are dynamically regulated by multiple binding (GlyR, GABARAP) and phosphorylation sites (via Erk1/2, GSK-3, Cdk5; Petrini and Barberis, 2014; Choii and Ko, 2015; Chapdelaine et al., 2021) and that gephyrin assembles into filaments that underlie phase separation to allow for flexible rearrangement of postsynaptic structures rather than forming a rigid lattice as previously believed (Macha et al., 2025). Thus, gephyrin mobility appears to be more dynamic than originally appreciated. These observations challenge the traditional view that receptor clustering is strictly secondary to scaffold formation and raise the possibility that, in some contexts, GABA_A_R clustering may precede or even initiate recruitment of mobile gephyrin assemblies to developing inhibitory synapses.

Because we observed that recruitment of gephyrin clusters to existing presynaptic boutons is proximity-dependent and occurs just prior to new colocalization events, we propose that local juxtacrine or paracrine signaling between Sema4D and Plexin-B1 guides the final trans-synaptic step after pre- and postsynaptic protein clusters are near each other. The molecular mechanisms linking Sema4D signaling to local recruitment of synaptic proteins remain largely unclear. Plexin-B1 is expressed both pre- and postsynaptically and is required in both compartments for Sema4D-dependent synapse formation (McDermott et al., 2018; Adel et al., 2024), but whether signaling acts simultaneously on both sides or whether one compartment signals to the other indirectly is unknown. Our finding that Sema4D-dependent changes to presynaptic mobility precede gephyrin localization to new contact sites implies that presynaptic effects are mediated by direct Sema4D binding to Plexin-B1 on the interneuron, while postsynaptic gephyrin mobilization occurs only once in proximity to a GAD65-positive bouton. This step could be mediated by paracrine Sema4D/Plexin-B1 interactions or by Plexin-B1 signaling in conjunction with coreceptors (Giordano et al., 2002; Swiercz et al., 2004).

Downstream signaling pathways activated by Plexin-B1 through the intracellular GAP domain and the C-terminal PDZ-RhoGEF binding motif mediate cytoskeletal remodeling (Ito et al., 2006; Tran et al., 2007; Vodrazka et al., 2009). One possibility is that Plexin-B1 directly promotes presynaptic protein mobility by regulating microtubule tracks, molecular motors, or force-generating cytoskeletal reorganization (e.g., actin branching or polymerization). A second possibility is that local cytoskeletal disruption or disassembly effectively “releases the brake” on presynaptic bouton mobility, allowing mobile boutons to sample a larger dendritic area, and stabilization subsequently occurs through contact with a postsynaptic specialization, preventing elimination. The latter hypothesis is supported by a study which demonstrated that local application of Sema4D to single boutons induced stabilization which could be chemically mimicked by destabilizing actin filaments (via latrunculin B treatment) or by inhibiting the RhoA/ROCK pathway (Frias et al., 2019). Although further work is required to distinguish between these possibilities, the relatively slow velocities and confined movement radii of protein clusters involved in new synapse formation point to modulation of local actin networks as the main structural change that promotes synapse formation downstream of Plexin-B1 signaling. Together, our data support a model in which Sema4D/Plexin-B1 signaling facilitates presynaptic protein mobility by removing actin-dependent constraints on preassembled presynaptic protein clusters and increasing the probability of transient contacts with nearby postsynaptic specializations which are then stabilized through reciprocal adhesion.

Overall, the findings from this study show that Sema4D signaling coordinates dynamic yet locally constrained changes in both pre- and postsynaptic compartments to assemble functional inhibitory synapses on rapid timescales. This capacity to precisely regulate inhibitory synapse formation has important implications for inhibitory circuit organization in the developing and mature brain: inhibitory synapses regulate the timing and synchrony of network activity, and disruptions to genes involved in inhibitory synapse assembly are implicated in various neurodevelopmental and seizure disorders (Shimojima et al., 2011; Sun et al., 2011; Lionel et al., 2013). The ability of Sema4D to coordinate pre- and postsynaptic protein mobility to rapidly assemble new synapses highlights a potential mechanism for fast, stable circuit remodeling and presents an intriguing yet largely unexplored therapeutic angle for disorders of excitatory–inhibitory balance such as epilepsy (Acker et al., 2018; Adel et al., 2023). More broadly, this model provides a general framework for how cellular signaling pathways may tune inhibitory connectivity and circuit balance on behaviorally and clinically relevant timescales.

## Synthesis

Reviewing Editor: Arianna Maffei, Stony Brook University Decisions are customarily a result of the Reviewing Editor and the peer reviewers coming together and discussing their recommendations until a consensus is reached. When revisions are invited, a fact-based synthesis statement explaining their decision and outlining what is needed to prepare a revision will be listed below. The following reviewer(s) agreed to reveal their identity: NONE. Note: If this manuscript was transferred from JNeurosci and a decision was made to accept the manuscript without peer review, a synthesis may not be available.

The authors have thoroughly addressed the reviewers' comments and substantially improved the manuscript with the addition of text edits, new analysis and extended data.
