# Supplementary figures and images for: Coordinated Pre- and Postsynaptic Protein Dynamics Underlie Rapid Sema4D-Induced Inhibitory Synapse Assembly
Source: eNeuro. 2026 May 29;13(6):ENEURO.0140-26.2026. doi: 10.1523/ENEURO.0140-26.2026 (PMC13225912; doi:10.1523/ENEURO.0140-26.2026)

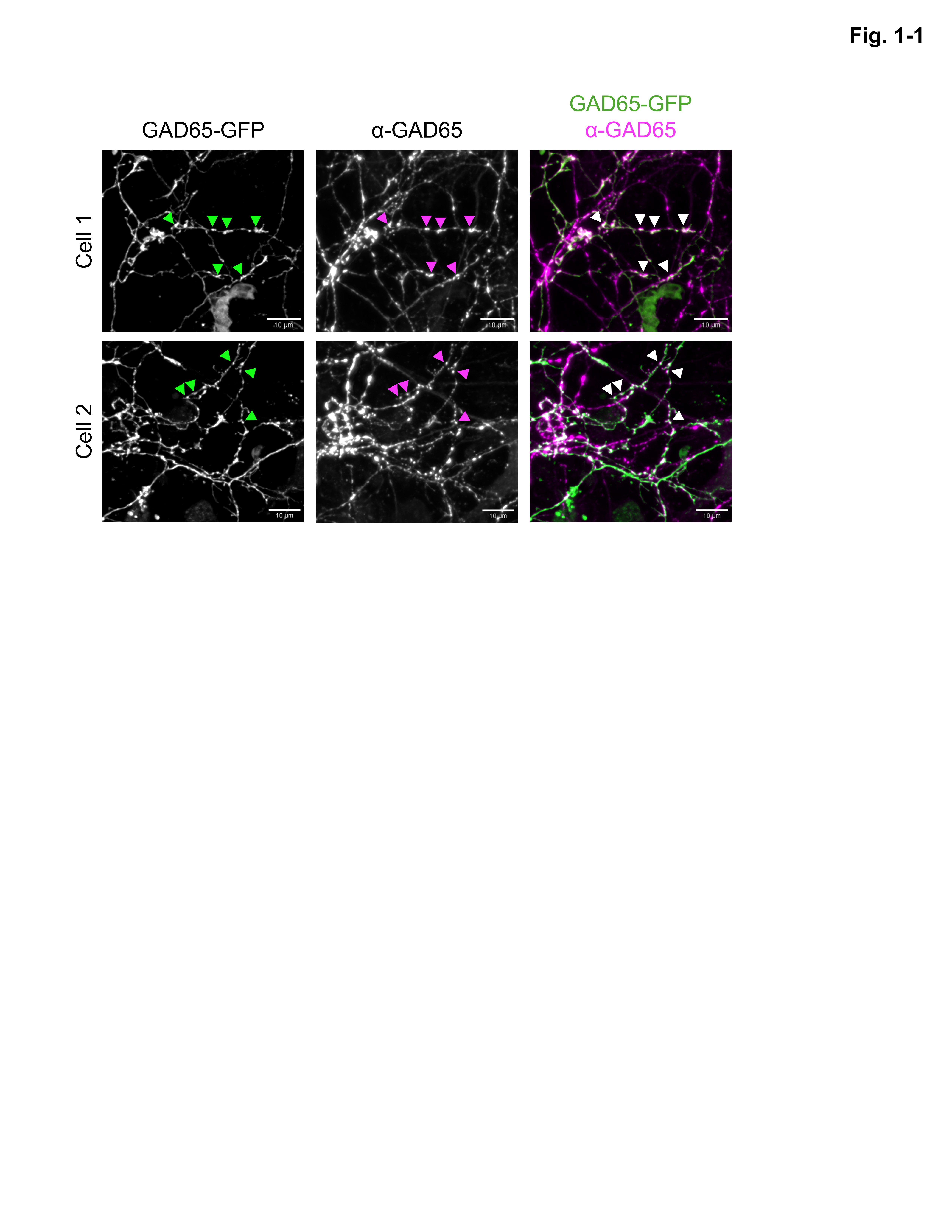

Supplement: Figure 1-1 — Boutons marked by GAD65-GFP are immunopositive for GAD65 protein. GAD65-GFP labeled boutons reliably colocalize with GAD65 antibody staining in distal axons of primary cultured DIV11 hippocampal neurons from GAD65-GFP mice. Green arrows = GAD65-GFP; magenta arrows = anti-GAD65. The majority of GAD65-GFP boutons in GFP-positive cells are marked by anti-GAD65 antibody (white arrows = colocalized). Scale bars = 10 µm. Download Figure 1-1, TIF file. [file eneuro-13-ENEURO.0140-26.2026-s001.tif]

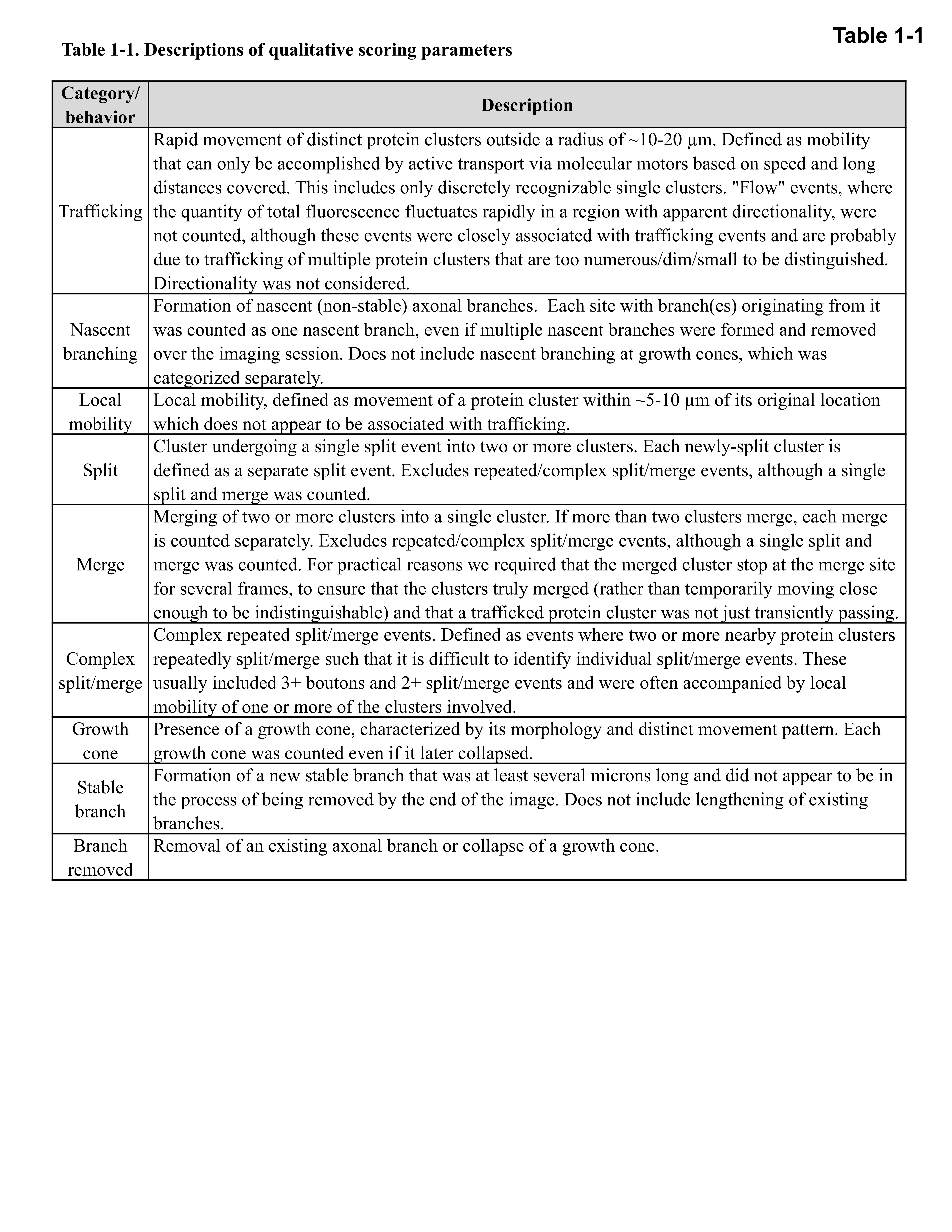

Supplement: Table 1-1 — Descriptions of qualitative scoring parameters. Download Table 1-1, TIF file. [file eneuro-13-ENEURO.0140-26.2026-s006.tif]

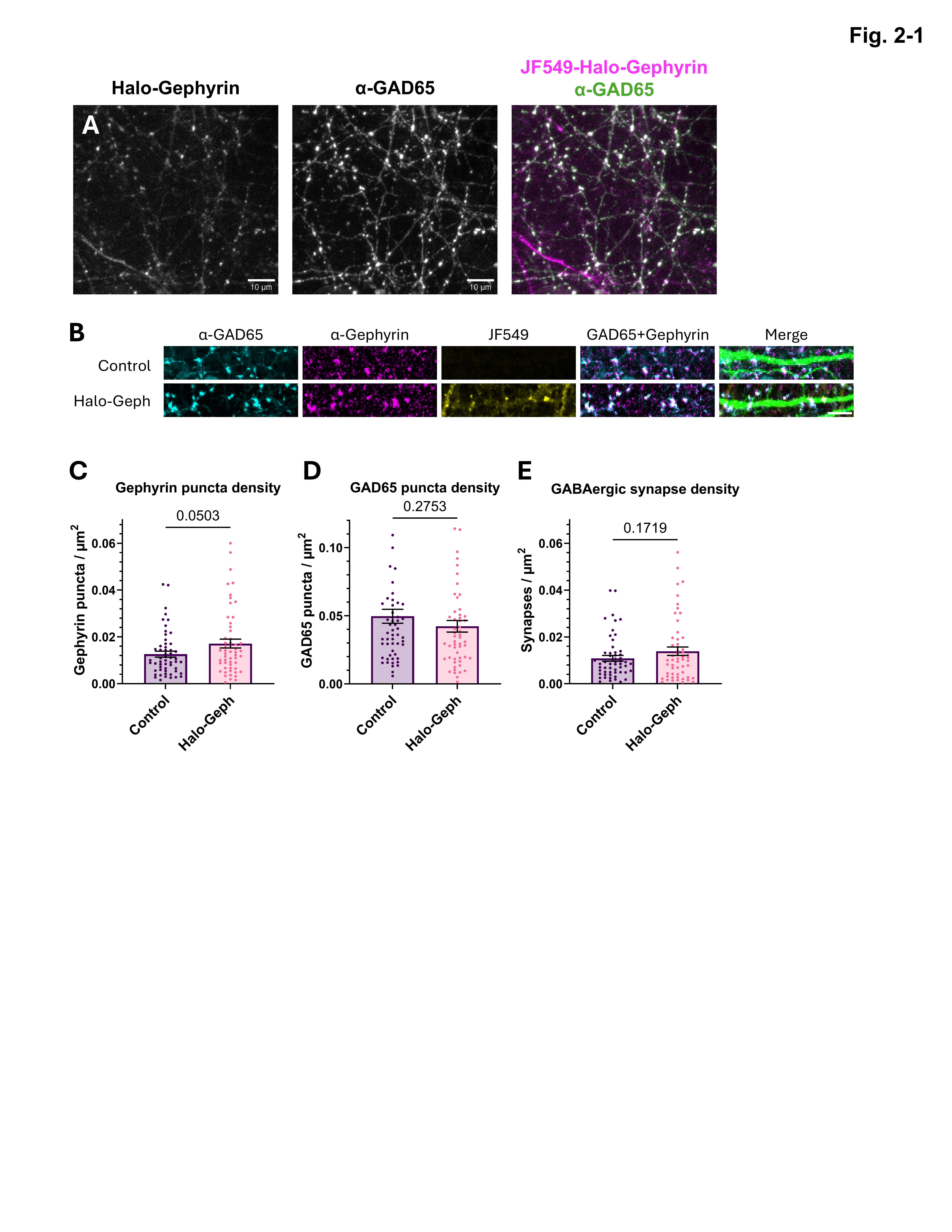

Supplement: Figure 2-1 — Viral expression of Halo-Gephyrin does not increase GABAergic synapse density. (A) Virally-expressed Halo-Gephyrin in cultured DIV11 rat neurons colocalizes with anti-GAD65 antibody. Magenta = Halo-Gephyrin, green = anti-GAD65 antibody, white = colocalized. Scale bar = 10 µm. (B) Sample stretches of dendrite from DIV11 rat neurons expressing GFP (green) with or without Halo-Gephyrin. Scale bar = 5 µm. (C) Viral Halo-Gephyrin expression marginally increases gephyrin puncta density compared to no-virus control neurons (unpaired t-test, p = 0.0503). n = 55 neurons from 2 replicates per condition. (D) Viral Halo-Gephyrin expression does not affect GAD65 puncta density compared to no-virus control neurons (unpaired t-test, p = 0.2753). n = 55 neurons from 2 replicates per condition. (E) Viral Halo-Gephyrin expression does not affect colocalized GAD65/gephyrin synapse density compared to no-virus control neurons (unpaired t-test, p = 0.1719). n = 55 neurons from 2 replicates per condition. Download Figure 2-1, TIF file. [file eneuro-13-ENEURO.0140-26.2026-s002.tif]

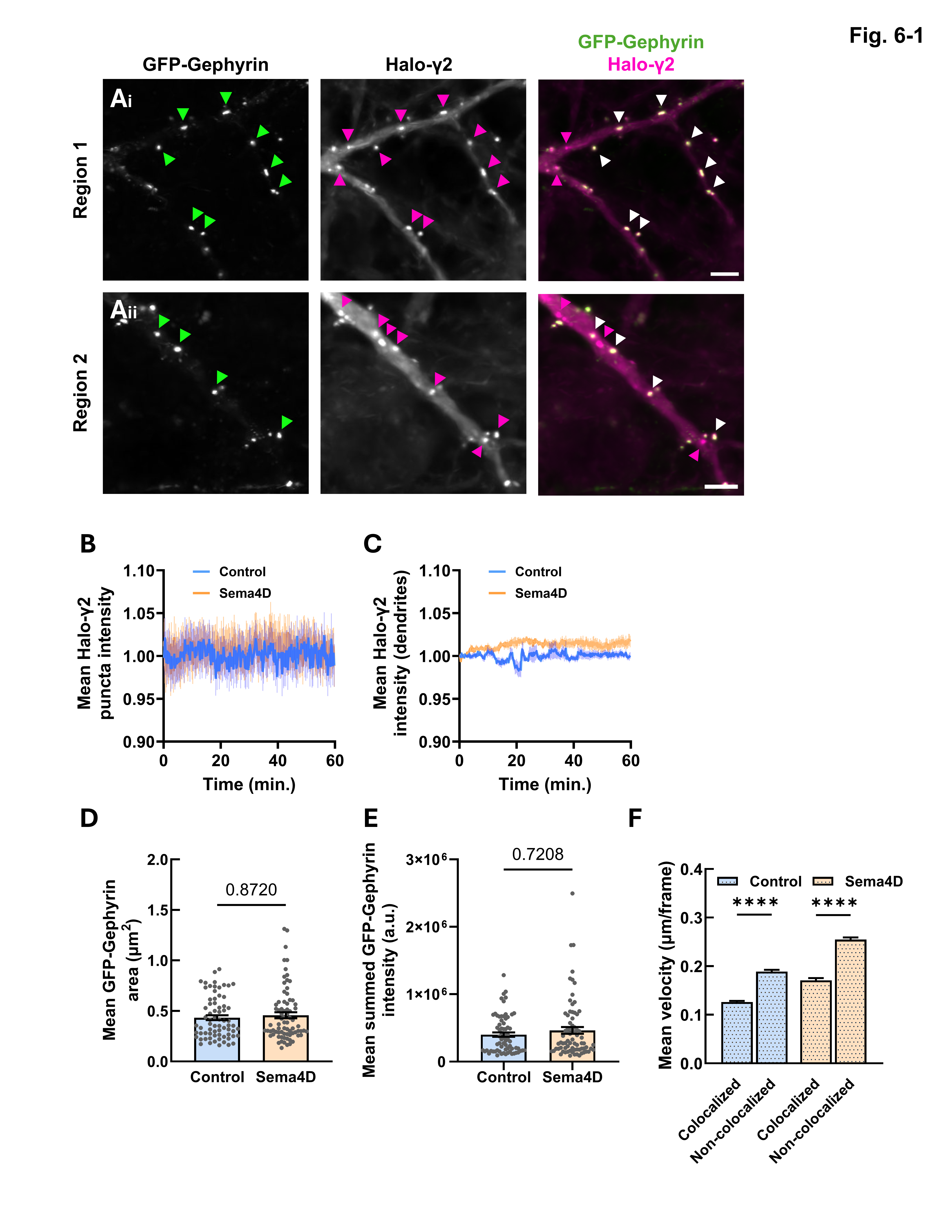

Supplement: Figure 6-1 — Sema4D does not affect mean Halo-γ2 puncta intensity or total dendritic intensity or GFP-Gephyrin size or intensity; independent Halo-γ2 puncta are more mobile than scaffold-associated puncta. (A) Representative regions showing expression pattern of GFP-Gephyrin (green) and Halo-γ2 (magenta) along dendrites of cultured E18 rat neurons. Most Halo-γ2 clusters are colocalized with GFP-Gephyrin (white arrows), but some independent clusters are observed (magenta arrows). Note diffuse extrasynaptic expression of Halo-γ2 along dendrite. Images represent different regions from the same neuron. Scale bar = 5 µm. (B) There was no effect of Sema4D treatment on mean intensity of Halo-γ2 puncta (binned LME: time × treatment interaction: F(1, 1508) = 0.0017 , p = 0.9670). n ≥ 69 puncta per timepoint (control), 45 puncta (Sema4D). Error bars = SEM. Data are normalized within treatment condition to the mean of the first 3 minutes. (C) There was no effect of Sema4D treatment on mean total dendritic expression of Halo-γ2 (binned LME: time × treatment interaction: F(1, 140) = 1.3144 , p = 0.2536). n = 7 cells (control), 5 cells (Sema4D). Error bars = SEM. (D) Sema4D does not affect mean area of GFP-Gephyrin that recruit Halo-γ2 compared to control (Mann-Whitney U-test, p = 0.8720). n = 70 puncta (control), 76 puncta (Sema4D). (E) Sema4D does not affect mean summed intensity of GFP-Gephyrin that recruit Halo-γ2 compared to control (Mann-Whitney U-test, p = 0.7208). n = 70 puncta (control), 76 puncta (Sema4D). (F) Mean velocity is significantly greater for Halo-γ2 puncta that are not colocalized with GFP-Gephyrin in both control (***p < 0.001, unpaired heteroscedastic t-test) and Sema4D-treated neurons (***p < 0.001). Download Figure 6-1, TIF file. [file eneuro-13-ENEURO.0140-26.2026-s003.tif]

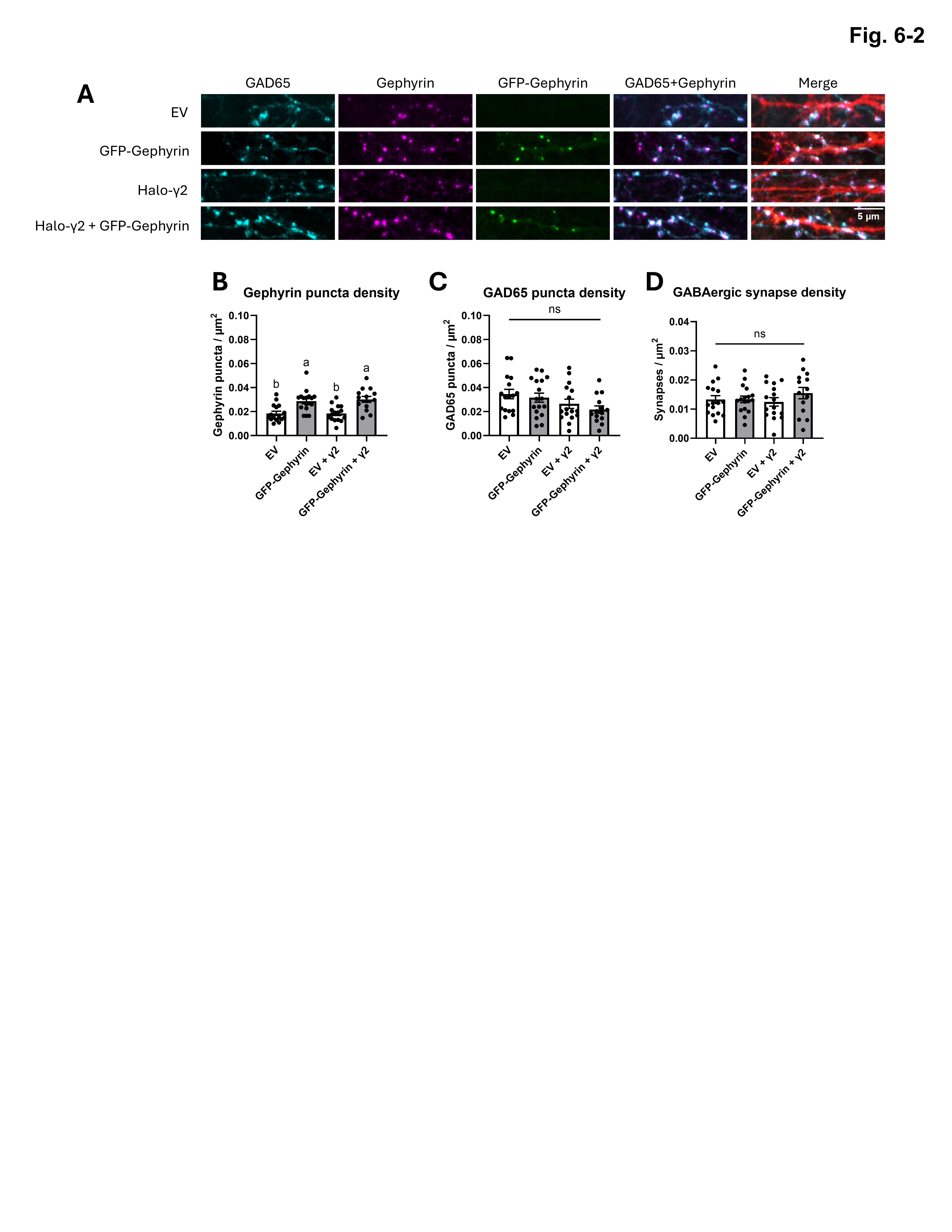

Supplement: Figure 6-2 — Overexpression of GFP-Gephyrin increases gephyrin puncta density but does not affect GABAergic synapse density; Halo-γ2 overexpression does not affect gephyrin puncta or synapse density. (A) Sample stretches of dendrite from neurons co-expressing TdTomato (red) with indicated constructs. Scale bar = 5 µm. (B) GFP-Gephyrin overexpression (OE) increases gephyrin puncta density on cultured neurons compared to empty vector (EV) control transfection (p = 0.0027, Tukey post-hoc), whereas Halo-γ2 OE does not affect gephyrin puncta density (p > 0.99). Co-expression of GFP-Gephyrin and Halo-γ2 does not increase gephyrin puncta density more than GFP-Gephyrin alone (p = 0.95). (C) Overexpression of GFP-Gephyrin alone, Halo-γ2 alone, or combined overexpression of GFP-Gephyrin + Halo-γ2 does not affect density of GAD65 puncta (F(3, 59) = 2.256, p = 0.0912). (D) Overexpression of GFP-Gephyrin alone, Halo-γ2 alone, or combined overexpression does not affect GAD65/gephyrin synapse density compared to EV control transfection (F(3, 59) = 1.676, p = 0.536, ordinary one-way ANOVA). Download Figure 6-2, TIF file. [file eneuro-13-ENEURO.0140-26.2026-s004.tif]

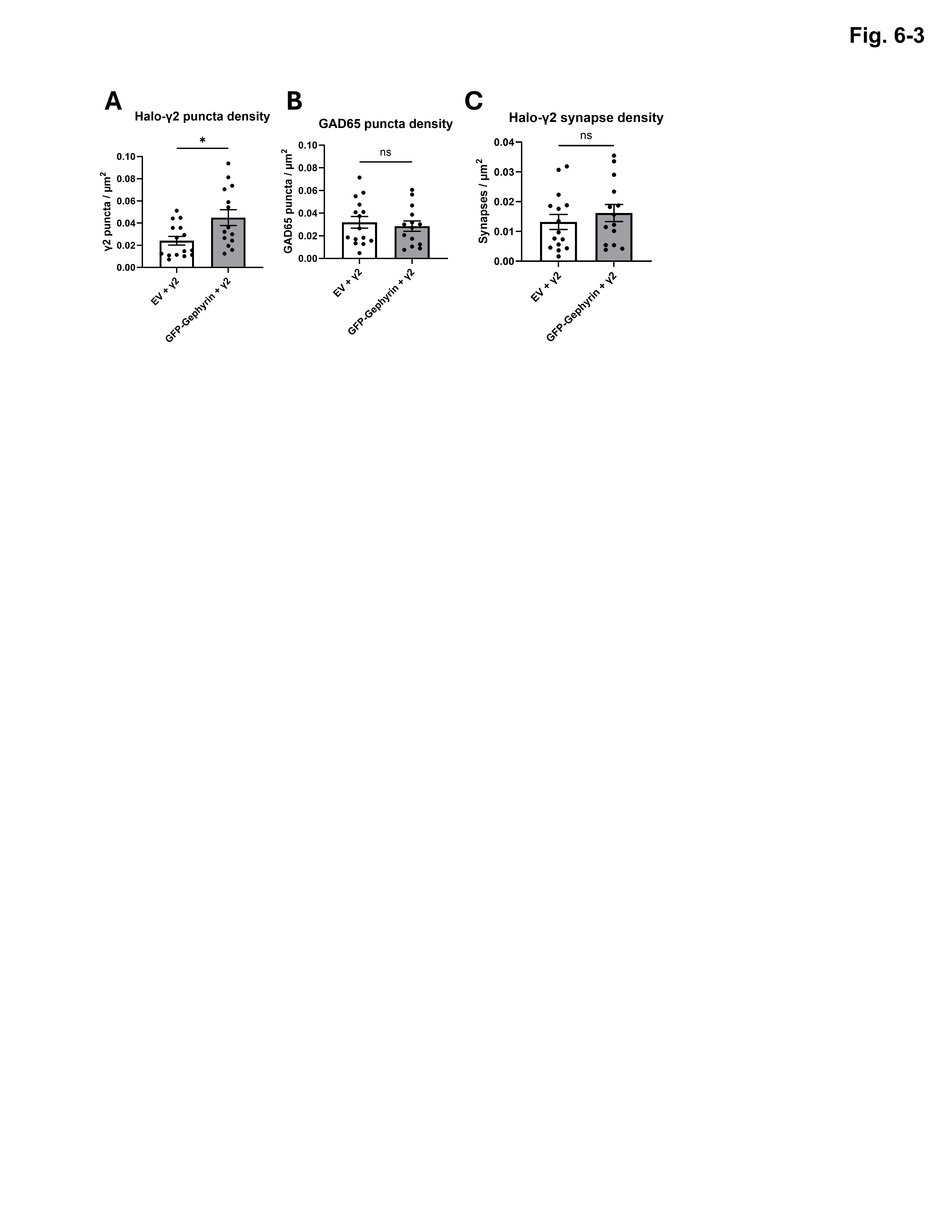

Supplement: Figure 6-3 — GFP-Gephyrin overexpression does not affect synaptic localization of Halo-γ2. (A) Co-expression of GFP-Gephyrin + Halo-γ2 increases the density of Halo-γ2 compared to Halo-γ2 OE alone (p = 0.0143, unpaired t-test). (B) GFP-Gephyrin OE does not affect density of GAD65+ inputs to transfected cells (p = 0.6309). (C) Co-expression of GFP-Gephyrin and Halo-γ2 does not affect Halo-γ2+ synapse density compared to Halo-γ2 OE alone (p = 0.436). Download Figure 6-3, TIF file. [file eneuro-13-ENEURO.0140-26.2026-s005.tif]
